# Nucleolar stress controls mutant Huntington toxicity and monitors Huntington’s disease progression

**DOI:** 10.1038/s41419-021-04432-x

**Published:** 2021-12-08

**Authors:** Aynur Sönmez, Rasem Mustafa, Salome T. Ryll, Francesca Tuorto, Ludivine Wacheul, Donatella Ponti, Christian Litke, Tanja Hering, Kerstin Kojer, Jenniver Koch, Claudia Pitzer, Joachim Kirsch, Andreas Neueder, Grzegorz Kreiner, Denis L. J. Lafontaine, Michael Orth, Birgit Liss, Rosanna Parlato

**Affiliations:** 1 Institute of Applied Physiology, Ulm University, Ulm, Germany; 2 RNA Molecular Biology, Fonds de la Recherche Scientifique (F.R.S./FNRS), Université Libre de Bruxelles (ULB), Biopark campus, Gosselies, Belgium; 3 Institute of Anatomy and Cell Biology, Heidelberg University, Heidelberg, Germany; 4 Division of Biochemistry, Mannheim Institute for Innate Immunoscience (MI3), Medical Faculty Mannheim, Heidelberg University, Mannheim and Center for Molecular Biology of Heidelberg University (ZMBH), DKFZ-ZMBH Alliance, Heidelberg, Germany; 5 Department of Medical-Surgical Sciences and Biotechnologies, University of Rome “Sapienza”, Rome, Italy; 6 Department of Neurology, Ulm University, Ulm, Germany; 7 Interdisciplinary Neurobehavioral Core (INBC), Heidelberg University, Heidelberg, Germany; 8 Maj Institute of Pharmacology, Department of Brain Biochemistry, Polish Academy of Sciences, Krakow, Poland; 9 Linacre & New College, University of Oxford, Oxford, UK; 10 Division for Neurodegenerative Diseases, Department of Neurology, Mannheim Center for Translational Neuroscience, Medical Faculty Mannheim, Heidelberg University, Mannheim, Germany

**Keywords:** Cell biology, Huntington's disease

## Abstract

Transcriptional and cellular-stress surveillance deficits are hallmarks of Huntington’s disease (HD), a fatal autosomal-dominant neurodegenerative disorder caused by a pathological expansion of CAG repeats in the Huntingtin (*HTT)* gene. The nucleolus, a dynamic nuclear biomolecular condensate and the site of ribosomal RNA (rRNA) transcription, is implicated in the cellular stress response and in protein quality control. While the exact pathomechanisms of HD are still unclear, the impact of nucleolar dysfunction on HD pathophysiology in vivo remains elusive. Here we identified aberrant maturation of rRNA and decreased translational rate in association with human mutant Huntingtin (mHTT) expression. The protein nucleophosmin 1 (NPM1), important for nucleolar integrity and rRNA maturation, loses its prominent nucleolar localization. Genetic disruption of nucleolar integrity in vulnerable striatal neurons of the R6/2 HD mouse model decreases the distribution of mHTT in a disperse state in the nucleus, exacerbating motor deficits. We confirmed NPM1 delocalization in the gradually progressing zQ175 knock-in HD mouse model: in the striatum at a presymptomatic stage and in the skeletal muscle at an early symptomatic stage. In Huntington’s patient skeletal muscle biopsies, we found a selective redistribution of NPM1, similar to that in the zQ175 model. Taken together, our study demonstrates that nucleolar integrity regulates the formation of mHTT inclusions in vivo, and identifies NPM1 as a novel, readily detectable peripheral histopathological marker of HD progression.

## Introduction

Dysregulation of rRNA biogenesis represents an emerging mechanism in several progressive neurodegenerative diseases characterized by proteinopathy [1–6]. Ribosomal RNA synthesis in the nucleolus—the most prominent nuclear compartment and a multilayered bio-molecular condensate—is tightly linked to the cell wellbeing, and it is highly responsive to cellular stress [7, 8]. Nucleolar stress is a p53-dependent anti-tumoral surveillance pathway activated upon ribosome-biogenesis dysfunction [9]. The shape of the nucleolus, its size, and the number of nucleoli per nucleus may change upon stress and in disease, reflecting changes in its function [10]. These properties have started to be explored as disease biomarkers [11, 12].

Huntington’ disease (HD) is caused by the expansion of CAG repeats in exon 1 of the Huntingtin (*HTT*) gene [13]. This autosomal dominant mutation results in an abnormal polyglutamine expansion in the Huntingtin protein with toxic effects [14]. Typical clinical hallmarks include motor, cognitive, and psychiatric symptoms [15]. Dopaminoceptive medium spiny neurons (MSNs) of the striatum are particularly vulnerable to neurodegeneration, along with reduced connectivity in regional and whole-brain cortico-caudate networks that highly correlate with cognitive and motor deficits [16]. Other non-neuronal features include metabolic and immune problems, malfunction of skeletal muscle, and body-weight loss [17].

The length of the expanded CAG tract in the mutant *HTT* gene partially accounts for the variability in the clinical HD onset [18]. Multiple pathophysiological mechanisms may contribute to HD [19]. Mutant HTT (mHTT) protein forms nuclear and cytoplasmic inclusions that interfere with almost all aspects of cell physiology, from nuclear transcription dysregulation to mitochondrial dysfunction, and compromised quality-control mechanisms, among many others [20, 21].

Previous studies showed that mHTT interferes with rDNA transcription and with the integrity of the nucleolus [3, 22–27]. In the striatum of the R6/2 transgenic mice, the *de novo* transcription of rRNA is impaired [26]. Several mechanisms have been proposed to explain how mHTT affects rRNA transcription [24, 25, 27]. mHTT protein acts on the acetyltransferase CBP (CREB-binding protein), required for the activity of the RNA polymerase I (RNA Pol I) [3, 24]. Moreover, *mHTT* mRNAs down-regulate rRNA transcription by interacting with the nucleolar protein nucleolin (NCL), that plays multiple roles in rRNA synthesis, ribosome biogenesis and nucleolar structure maintenance [25, 28, 29]. PGC-1alpha (peroxisome proliferator-activated receptor gamma coactivator 1alpha), a master regulator of mitochondrial biogenesis, which is transcriptionally repressed by mHTT, also controls rDNA transcription in the nucleolus [27]. Importantly, brain derived neurotrophic factor (BDNF) known to sustain striatal neuron survival and downregulated in HD [30, 31], stimulates the activity of the transcription-initiation factor-IA (TIF-IA), essential for the recruitment of the RNA Pol I at the ribosomal promoters [32].

The nucleolus is also involved in protein quality control to prevent the irreversible aggregation of misfolded proteins, a mechanism often altered in several aggregate-forming neurodegenerative diseases [33, 34]. In particular, the nucleolar protein nucleophosmin-1 (NPM1) appears to have a chaperone-like function in shielding the surfaces of potentially toxic aggregates [34].

Despite the multiple relationships between transcriptional, metabolic, and quality-control functions of the nucleolus and HD pathophysiology, the impact of nucleolar dysregulation on HD progression in vivo has not been systematically addressed. It also remains unexplored whether different disease stages are associated with context-specific changes in nucleolar transcription and integrity. Ultimately, cytomorphological nucleolar alterations related to HD pathophysiology in peripheral tissues might represent novel metabolic markers to monitor disease progression and treatment response.

To gain insight into the mechanistic relationship between nucleolar dysfunction and disease progression, we investigated the consequences of nucleolar stress on mHTT inclusions and motor symptoms in R6/2 mice. We used a functional genomic approach to inhibit RNA Pol I transcriptional activity in vulnerable striatal neurons of these mice. We have previously shown that the conditional ablation of *Tif-Ia* in MSNs of the striatum that express the dopamine 1 receptor (D1R) mimics a condition of progressive nucleolar stress in vivo [26]. Given that TIF-IA is targeted by several kinase cascades and integrates multiple signaling pathways, it represents an ideal molecular target to regulate rDNA transcription in a cell-type-specific fashion [35, 36].

## Materials and methods

### Statement regarding the ethical use of human material and animals

The ethics committee at Ulm University approved the study (Protocol number: 165/12), and written informed consent was obtained from each participant.

Procedures involving animal care and use were approved by the Committee on Animal Care and Use (Regierungspräsidium Karlsruhe, Germany, Animal Ethic Protocols: 34-9185.81/G-297/14 and 35-9185.81/G-102/16) in accordance with the local Animal Welfare Act and the European Communities Council Directives (2012/707/EU).

### Human skeletal muscle biopsies

Ten *HTT* CAG trinucleotide repeat expansion carriers and five healthy sex- and age-matched controls were recruited at the Department of Neurology of Ulm University (Table S1). Participants had no contra-indications to muscle biopsy, e.g., a clotting disorder or abnormalities on electrocardiograms. *HTT* CAG repeat length was determined and participants were clinically assessed as described for the TrackOn and TRACK-HD studies [37, 38]. The disease burden score (DBS) was calculated from each HD participant’s CAG repeat length and age according to the following formula: (CAG-35.5) × age [39]. Clinical assessment included the United Huntington’s Disease Rating Scale (UHDRS) motor part to derive the total motor score (TMS) [13] and the UHDRS total functional capacity scale (TFC). Potential HD participants were screened if they had a disease-burden score of 250 or greater and either had no clinical signs of manifest motor HD (preHD) or were in TFC stages of 1 or 2 indicative of early motor manifest HD (earlyHD). Table S1 summarizes the participant demographic, number of CAG repeat, disease burden, and UHDRS total motor-score data.

Open biopsies of the *M. vastus lateralis* were obtained following local anesthesia. For immunofluorescence muscle was mounted on a piece of cork in TissueTek (Sakura Finetek, Germany) with fibers oriented perpendicular to the cork and then snap-frozen in liquid N_2_-cooled 2-methylbutane and stored at −80 °C until sectioning.

### Mice

B6CBA-Tg(HDexon1)62Gpb/1 J (R6/2) transgenic mice (CAG160) were imported from the Jackson Laboratories (Bar Harbour, ME, USA). Htt^tmtm1Mfc^/190tChdi (zQ175 knock-in) mice (CAG198) were received from the CHDI Foundation by the Jackson Laboratories. For the experiments reported here, male and female mice were used, and wild-type and mutant littermates were analyzed. The zQ175 knock-in mice carry ca. 190 CAG repeats in a chimeric human/mouse exon 1 of the murine *Htt* gene [40]. The zQ175 mutation was kept in heterozygosity, to limit toxicity and mimic the most common genetic condition in HD [40]. The conditional knockout of the nucleolar transcription factor *Tif-Ia* gene by the Cre-LoxP system in D1R-expressing cells (official nomenclature: B6.129.FVB/N-TIF-IA^tmGSc^Tg(D1RCre)^GSc^, abbreviated as TIF-IA^D1Cre^) was achieved as previously described [26]. Mice were housed in a standard 12-h light/dark cycle and kept with ad libitum access to food and water. The analysis of the genotype was performed by PCR of tail snips as previously described [26, 41, 42]. Randomization was not applicable.

### Tissue processing for RNA extraction and quantitative real-time PCR (qRT-PCR) in mice

Total RNA was isolated from dissected mouse striatum in the region comprised between Bregma 1.34 mm and −0.34 mm [43]. Synthesis of cDNA with M-MLV Reverse Transcriptase (SuperScript III First Strand Synthesis Supermix, 18080-400, Thermo Scientific, Waltham, USA) was primed with random hexamers. For detection of pre-rRNA, either the first 130 nucleotides relative to the transcription start site (47 S 1) were amplified or a primer pair covering the first processing site (47 S 2) was used, as previously described (Kiryk et al, 2013). The TaqMan gene expression assays (ThermoFisher Scientific) used for this study are reported in Table S2. Pre-rRNA expression in the striatum was normalized using the stably expressed reference gene *Gapdh*, while for the TaqMan assays, we used the stably expressed *Hprt* (hypoxanthine–guanine phosphoribosyl transferase) for striatal tissue and *Metap1* (methionine aminopeptidase 1) for muscle tissue [44, 45]. Changes in relative expression were calculated as a fold change versus mean of the respective control samples.

### Tissue processing for immunostaining in mouse and human tissues

Mice were sacrificed by cervical dislocation and brains and skeletal muscle (quadriceps) were immediately dissected. For immunofluorescence and immunohistochemistry, sectioning and immunostaining was performed, as reported in the Supplementary Materials and Methods.

### Mouse cell tissue culture conditions

The StHdh Q7/Q7, Q7/Q111, and Q111/Q111 cells (striatal neuronal derived from Hdh7 wild-type and Hdh111 knock-in mice) [23] were cultured as previously described [42]. Mycoplasma/microbial contamination was tested regularly.

### Pre-rRNA processing analysis

Total RNA from the StHdh Q7/Q7, Q7/Q111, and Q111/Q111 cells was extracted, and processed for Northern blotting according to [46]. The probe used was LD4098 (5′-ETS_A0-1, ACAATGACCACTGCTAGCCTCTTTCCCTT).

### Confocal and STED super-resolution microscopy

Confocal images of the striatum and muscle sections were obtained as z-stacks using a confocal scanning microscope (Leica TCS SP8) with a 63X/1.32NA oil objective and Leica LAS X imaging software (Leica Microsystems, Wetzlar, Germany). STED images were acquired as z-stacks in 0.5 µm steps at the Stedycon (Abberior Instruments, Göttingen, Germany) with a 100X/1.4NA oil objective. STED images were deconvoluted by the Huygens software (Scientific Volume Imaging, Hilversum, The Netherlands).

### Image analysis

All microscopic images were analyzed by an experimenter blinded to the genotype and age using the Fiji software [47]. The number and area of nucleolar markers were analyzed, as described in Supplementary Methods and in [26, 48–50].

### Statistics

Mean values per sample were used for statistical analysis (Graphpad Prism 7.04 software). Datasets were analyzed for their statistical significance using nonparametric unpaired, two-tailed Mann–Whitney U (MWU) test or using the Kruskal–Wallis test followed by Dunn’s post hoc analysis for multiple comparisons. For all tests statistical significance level was set at p < 0.05. Complete details about statistical analysis are provided as Supplementary Statistical Information.

## Results

### Expression of the human Huntingtin polyQ111 mutation alters NPM1 localization, pre-rRNA processing, and translation

**Fig. 1:**
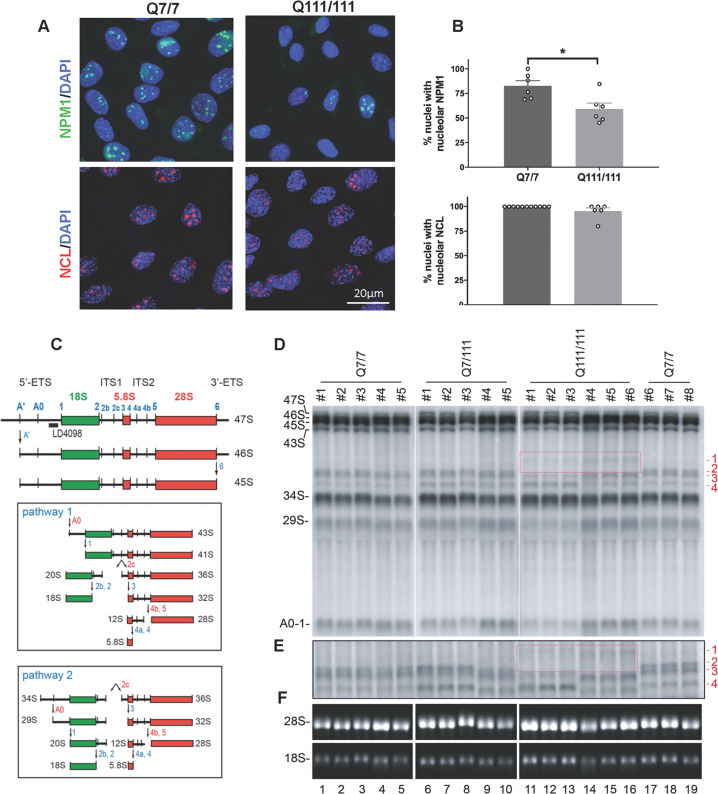
Cells expressing Huntingtin mutations display altered distribution of nucleolar NPM1 and alterations of pre-rRNA processing. **A** Representative confocal images of NPM1 and NCL immunofluorescence staining in Q7/7 and Q111/111 cells. **B** Quantification of the percentage of nuclei with nucleolar ring-like NCL and NPM1 signals. The percentage of nuclei with nucleolar NPM1 is significantly reduced in the Q111/111 cells by Mann–Whitney U test (*p* = 0.014) (n: number of nuclei = 421 in Q7/7 and 634 in Q111/111; N: fields of view in two independent experiments = 6 for Q7/7 and Q111/111). Values represent mean values and error bars are SEM. *p < 0.05. Detailed statistical information is included in the Supplementary Statistical Information file. **C** Mouse pre-rRNA processing pathway. Three out of four mature rRNAs (the 18 S, 5.8 S, and 28 S) are encoded in a single polycistronic transcript synthesized by RNA polymerase I, the 47 S. Mature rRNAs are embedded in 5′ and 3′ external transcribe spacers (5′- and 3′-ETS) and internal transcribed spacers 1 and 2 (ITS1 and 2) and are produced by extensive processing of the 47 S. Processing sites (A′, A0, 1 etc.) are indicated in blue. There are two alternative processing pathways in mouse (pathways 1 and 2, boxed) according to initial processing in 5′-ETS or ITS1, respectively. **D** Total RNA was extracted, separated on denaturing high-resolution agarose gel, stained with ethidium bromide to reveal large mature rRNAs, or processed for Northern blotting. Species labeled “1–4” (in red) are extended forms of the 34 S pre-rRNAs that were not previously described. Species “2, 3, and 4” are detected in the control cells (Q7/7). Species “1” is only detected in the mutant cells (Q111/Q111). Species “1” is formed at the cost of species “2” (the upper band of the doublet). The boxed area highlights the appearance of species “1” in Q111/Q111 cells, which is concomitant with the disappearance of species “2”. **E** The samples described in panel (**D**) were run in a longer migration to separate more efficiently the doublet corresponding to bands “2 and 3”. **F** Ethidium bromide staining revealing that the steady state levels of 18 S and 28 S rRNA are not grossly affected.

To investigate the functional impact of mHTT expression on ribosome biogenesis and function, we analyzed a mouse cell model of HD derived from embryonic striatum and expressing a chimeric human-mouse mutant Huntingtin [23]. The StHdhQ111/Q111 cells (abbreviated “Q111/111”) contain the polyQ111 mutation encoded by the CAG expansion on both alleles in the Huntingtin gene. As control, we used Q7/Q7 cells that express a nonpathological 7-glutamine wild-type protein on both alleles (Fig. 1). The localization of NPM1 present in the nucleolar granular component, where late steps of ribosomal subunit assembly occur, was reduced (ca. 30%) in the Q111/111 cells in comparison with the control (Fig. 1A, B). Importantly, NCL, another nucleolar protein, did not show any significant differences in abundance or distribution (Fig. 1A, B).

To test if mHTT expression impacts pre-rRNA processing, total RNA was extracted from cells expressing each construct, and separated on denaturing agarose gels. Precursor rRNAs were detected with specific radioactive labeled probes (Fig. 1C–E). We observed no gross alteration in the steady-state accumulation of the large mature rRNAs, the 18 S and 28 S rRNAs (Fig. 1F). The canonical pre-rRNA processing intermediates (29 S, 34 S, 43 S, 45 S, 46 S, and 47 S) also accumulated normally (Fig. 1D). However, close inspection of the imaging plates reproducibly revealed additional low-abundant pre-rRNA intermediates, referred to as “1”, “2”, “3”, and “4” **(**in red in Fig. 1D, E). Species “2”, “3” and “4” visible in the Q7/7 cells corresponded to extended forms of the 34 S pre-rRNA. Cells that expressed the Q111/Q111 mutation, showed a novel band, labeled as “1” (lanes 11–16, red box). Interestingly, when species “1” was detected, “2” was not, pointing to a precursor-product relationship that was specific to the Q111/111 cells. Remarkably, the cryptic species “1” was not observed in the heterozygous cells (Q7/Q111), indicating that sufficient amounts of Q111/111 mHTT must be expressed for processing to be altered.

To investigate whether the processing alterations impact translation, we performed polysomal analysis in the Q7/7 and Q111/111 cells (Fig. S1A, B). Velocity-gradient centrifugation allows to separate and to quantify free ribosomal subunits (40 S, 60 S), monosomes (80 S), and polysomes, and it is a good proxy of global protein synthesis. Polysomal profiles revealed a deficit in global translation, by the reduction of polysomal fraction in Q111/111 cells, while the number of ribosomal subunits was similar to that of Q7/7 cells (Fig. S1A, B). This effect on translation was confirmed in Q111/111 cells by use of the SUrface SEnsing of Translation (SUnSET) assay that monitors translation rates by labeling nascent proteins with the amino-acid analog puromycin (Fig. S1C, D).

In summary, expression of mHTT was associated with the specific loss of NPM1 nucleolar localization, the activation of cryptic pre-rRNA processing, alterations in RNA cleavage kinetics, and reduced global translation.

### Nucleolar stress exacerbates mHTT neuropathology and motor-behavior deficits in the R6/2 model of HD

**Fig. 2:**
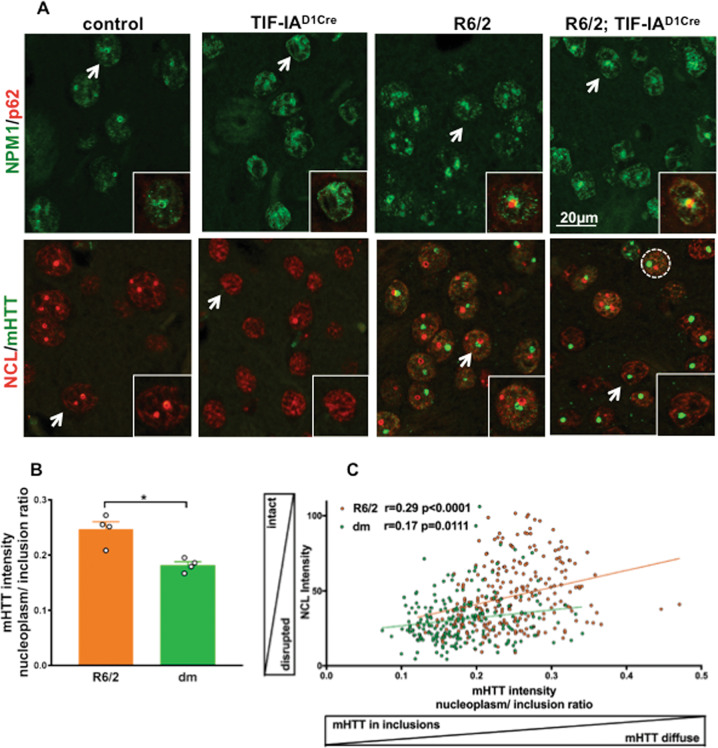
Delocalization of NPM1 and NCL and altered nuclear distribution of mHTT inclusions upon irreversible induction of nucleolar stress in striatal neurons. **A** Representative confocal images of striatal sections from control, TIF-IA^D1Cre^, R6/2 and R6/2; TIF-IA^D1Cre^ double mutant (dm) mice at 9 weeks co-immunostained with antibodies against the nucleolar marker NPM1 and NCL, and p62 and mHTT (EM48), respectively. Insets are high magnification of the nuclei highlighted by the white arrow. Scale bar: 20 µm, 10 µm (insets). **B** mHTT nuclear distribution measured as the ratio between mHTT mean intensity in the nucleoplasm and in the nuclear inclusions in R6/2 (N = 4) and dm (N = 4) mice. mHTT intensity in the nucleoplasm of dm is significantly lower in comparison with R6/2 mice (Mann–Whitney U test, *p* = 0.03). **C** Higher nucleolar integrity assessed by NCL intensity in the nucleus correlates with a higher nucleoplasm/inclusion mHTT intensity ratio in R6/2 (n: number of nuclei = 335, N: number of mice = 4) and dm mice (n: number of nuclei = 296, N: number of mice = 4); (*p* < 0.0001 for R6/2 and dm; Pearson coefficient r for R6/2 is 0.67 and for the dm 0.68).

To investigate the impact of nucleolar stress on HD neuropathology, we genetically inhibited nucleolar function in striatal MSNs of the R6/2 model (Fig. 2 and Figs. S2, 3). In particular, using the Cre-loxP system we conditionally ablated TIF-IA in dopamine-receptive neurons of R6/2 mice, and we generated double mutant R6/2; TIF-IA^D1Cre^ mice (abbreviated “dm”) (Fig. 2).

Confocal microscopy on striatal sections immunolabeled by NPM1 and NCL showed that in control mice at 9 weeks, the nucleolus was visible as a circular structure within the nucleus (Fig. 2A). At the same age in the TIF-IA^D1Cre^ mice, the percentage of DAPI labeled nuclei with NPM1 and NCL positive nucleoli was strongly decreased in comparison with control **(**Fig. S2A). In the R6/2 mice, both NPM1 and NCL maintained a ring-like organization, however NPM1 signal was diffuse **(**Fig. 2A and Fig. S2A). In particular, NPM1 surrounded the cargo protein p62/SQSTM1 in both the R6/2 and dm mice, suggesting a possible interaction with protein aggregates (Fig. 2A).

Next, we compared the accumulation and distribution of mHTT in the R6/2 and in the dm mice, by immunofluorescence using the EM48 antibody. As expected, mHTT was absent in striatal sections from control and TIF-IA^D1Cre^ mice (Fig. 2A). In the R6/2 and dm mice, mHTT was visible as intranuclear inclusions and scattered in the nucleoplasm (Fig. 2A). The number and size of intra-nuclear inclusions containing mHTT was comparable between R6/2 and the dm (Fig. S2B). To gain further insight into the mHTT nuclear distribution upon induction of nucleolar stress, we measured the ratio between the intensity of mHTT signal in the nucleoplasm and in the nuclear inclusion bodies. This ratio was about 30% lower in the dm mice, suggesting that nucleolar stress alters mHTT distribution (Fig. 2B). Disruption of nucleolar integrity determined by measuring nucleolin intensity in the nucleus in the R6/2 and in the dm mice, correlated with the changes in mHTT distribution detected by the mHTT intensity ratio **(**Fig. 2C). This loss of diffuse nucleoplasmic mHTT was supported by immunohistochemistry with the EM48 antibody (Fig. S2C–E).

To further investigate whether nucleolar stress results in a more severe HD-like phenotype, we examined behavioral paradigms related to HD in control, TIF-IA^D1Cre^, in R6/2 and in dm mice at 9, 10 and 11 weeks (Fig. S3A–E). At 9 weeks, the dm mice display about five times more clasping episodes than the controls and TIF-IA^D1Cre^, without significant increase in the dm compared with the R6/2 mice (Fig. S3B). At 10 weeks, the dm performed significantly worse than the R6/2 mice, showing a more severe deficit in coordinating gait changes with increasing acceleration (Fig. S3D). In line with these results, at 10 weeks the dm performed less well in the forelimbs grip strength test, in comparison with all other groups (Fig. S3E).

These results indicated that NPM1 and NCL are differentially perturbed, and the pattern of mHTT accumulation points to a more advanced neuropathology in the dm mice.

### Reduced nucleolar localization of NPM1 in the striatum of the late-onset zQ175 mouse model of HD

**Fig. 3:**
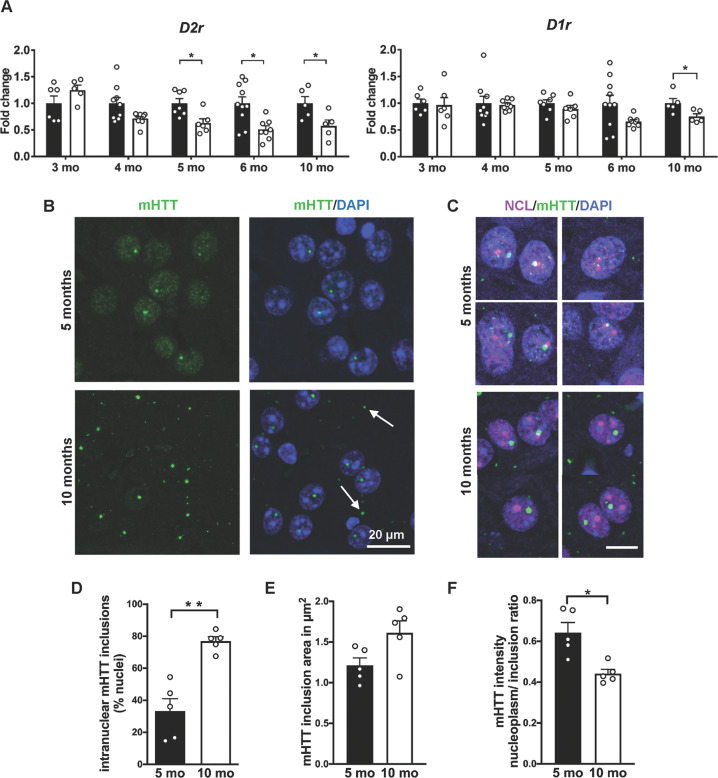
mHTT nuclear distribution changes at different stages in the striatum of zQ175 knock-in model. **A** Relative expression of *D2r* and *D1r* mRNA in the striatum by qRT-PCR at 3, 4, 5, 6 and 10 months (mo) in control (N = 6, 8, 7, 10, 5) and zQ175 mutant (N = 6, 8, 6, 8, 5) mice is expressed as fold change to the respective controls. Significantly decreased relative expression of *D2r* at 5 (*p* = 0.014), 6 (*p* = 0.012), and 10 months (*p* = 0.032) and of *D1r* at 10 months (*p* = 0.032) by Mann–Whitney U test. **B**, **C** Representative confocal images of striatal sections from zQ175 mice at 5 and 10 months stained with antibodies against mHTT (EM48) and counterstained with DAPI to visualize the nuclei, and with antibodies against mHTT (EM48) and NCL. Scale bar: 20 µm (**B**), 10 µm (**C**). **D** Quantification of the nuclei with mHTT inclusion bodies in the zQ175 mice at 5 and 10 months shows a significant increase at 10 months by Mann–Whitney U test (*p* = 0.008). **E** Nonsignificant statistical differences in the mean area of the mHTT inclusion signal by Mann–Whitney U test (*p* = 0.09) between zQ175 mice at 5 and 10 months. **F** Nuclear distribution of mHTT is measured as the ratio between mHTT mean intensity in the nucleoplasm and in the nuclear inclusions at 5 and 10 months. mHTT intensity in the nucleoplasm at 10 months is significantly lower by Mann–Whitney U test (*p* = 0.016). N: number of mice = 5; values represent mean values and error bars are SEM. *p < 0.05, **p < 0.01.

**Fig. 4:**
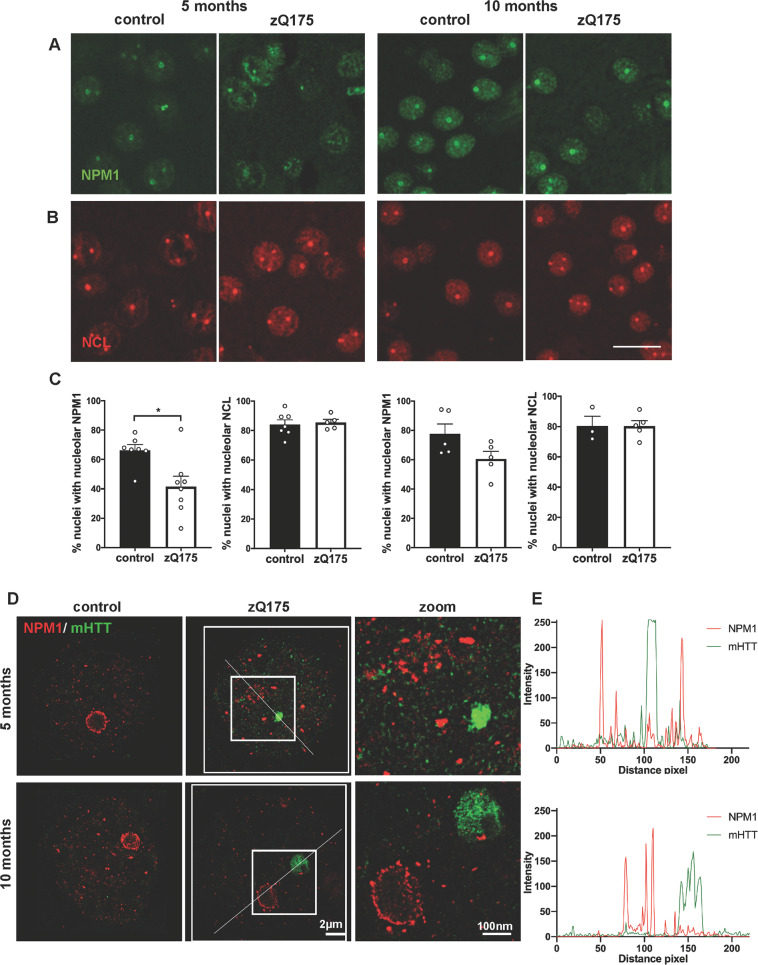
Redistribution of NPM1 in the striatum of pre-symptomatic zQ175 mice. **A**, **B** Representative confocal images of striatal sections stained for NPM1 (green) and NCL (red) in control and zQ175 mice at 5 and 10 months. Scale bar: 20 µm. **C** Quantification of the percentage of nuclei with nucleolar localization of NCL or NPM1 expressed as percentage of nuclei showing them as a circular nucleolar signal at 5 and 10 months in control and zQ175 mice (N: number of mice, control: 7,7,5,3 and zQ175: 8,5,5,5). Significant decrease in the number of nuclei showing nucleolar NPM1 in the zQ175 mice at 5 months in comparison with their respective controls by Mann–Whitney U test (*p* = 0.02). Values represent mean ± SEM. *p < 0.05. **D** Super-resolution (STED) microscopic images showing loss of NPM1 (red) ring-like organization and its association with disperse mHTT (green) signals at 5 months, but not at 10 months. **E** Line scans through the boxed regions containing the nucleolus and mHTT inclusion describe the distribution of NPM1 and mHTT signals in the zQ175 mice at both ages. A close proximity of mHTT and NPM1 signals at 5 months can be observed. Scale bar: 2 μm, zoom: 100 nm.

To monitor nucleolar activity and integrity in a gradually progressing model of HD, we analyzed heterozygous zQ175 knock-in mouse model of HD at pre-symptomatic (5 months) and symptomatic ages (10 months) [40, 51]. At 5 months, body weight and motor behavior of zQ175 heterozygous mice were comparable to that of control littermates (Fig. S4). In line with a more advanced disease stage, both dopamine 2-receptor *(D2r)* and D1-receptor (*D1r*) mRNAs were decreased at 10 months (Fig. 3A) [40]. The percentage of nuclei showing mHTT inclusions, area of the mHTT inclusions, and relative mHTT intensity in the nucleoplasm and in the inclusions confirmed the more advanced neuropathological stage at 10 months (Fig. 3B–F) and [51]. The analysis of human BA4 cortex RNAseq data (GSE79666) [52] and data from the striata and gastrocnemius of zQ175 mice at different ages (HDinHD.org) [53] shows dysregulation of genes involved in rRNA transcription and pre-rRNA processing in this model at about 6 months (Fig. S5). These observations along with the close interaction of mHTT with the nucleolus identified by immunofluorescence colocalization of NCL with mHTT in the zQ175 model (Fig. 3C), led us to investigate changes in NPM1 redistribution by confocal microscopy (Fig. 4). The number of DAPI positive nuclei showing a distinct NPM1 nucleolar staining was significantly reduced (ca. 40% less) in striatal neurons of zQ175 mice at 5 months (Fig. 4A, C). At 10 months, a ca. 20% reduction was observed, however it was not statistically significant (Fig. 4A, C). Similar to the Q111/111 cells and R6/2 mice, we found no evidence for change of NCL in the zQ175 mice (Fig. 4B, C). To further investigate the spatial distribution of NPM1 with respect to the mHTT nuclear foci, we performed STED microscopy in the striatum of zQ175 mice (Fig. 4D). We observed a close proximity between diffuse nucleoplasmic NPM1 and mHTT that appear intermingled at 5 months (Fig. 4D, **upper panels**), while at 10 months, mHTT was mostly in the nuclear inclusion body (Fig. 4D, **lower panels**), showing that redistribution of nucleolar NPM1 precedes the formation of large mHTT inclusions. By qRT-PCR assays, RNA in situ hybridization and Northern blots, we did not detect any significant differences in the 47 S pre-rRNA, and mature 18 S and 5.8 S rRNA at the two considered stages (Fig. S6A–G).

In summary, our results from striatal neurons in different models of HD identify nucleolar stress as a mechanism exacerbating disease progression, and NPM1 redistribution as a marker for disease progression and mHTT aggregation.

### Reduced nucleolar localization of NPM1 in skeletal muscle of zQ175 mice

**Fig. 5:**
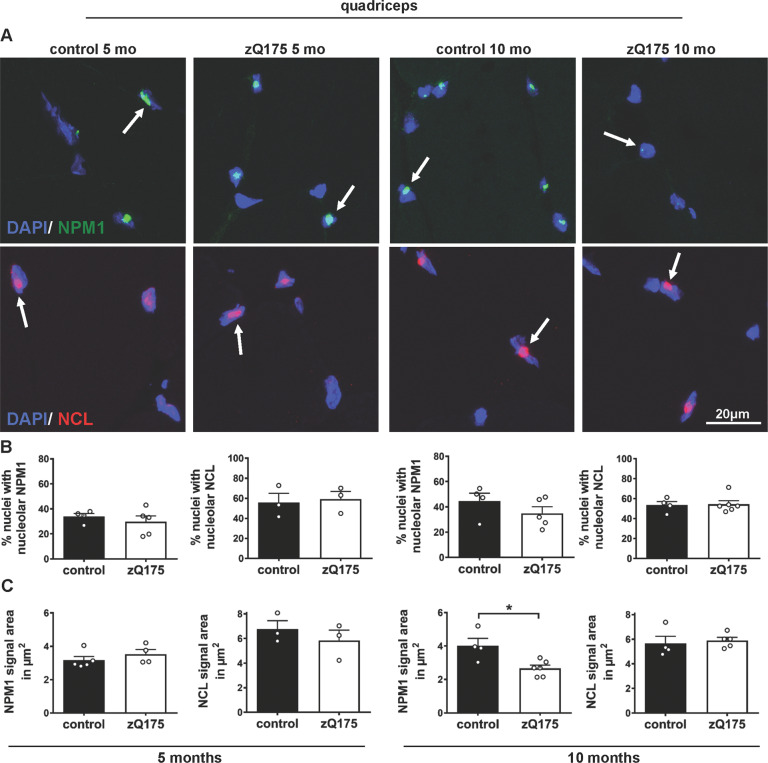
NPM1 nucleolar localization is altered in the skeletal muscle (quadriceps) of zQ175 mice at a symptomatic stage. **A** Representative confocal images of quadriceps cryosections stained for NPM1 (green) or NCL (red) in control and zQ175 mice at 5 and 10 months. Nuclei are labeled with DAPI (blue). The arrows point out to NPM1 or NCL signal. Scale bar: 20 μm. **B** Quantification of the percentage of nuclei with nucleolar localization of NPM1 or NCL at 5 and 10 months (mo) in control (N, number of mice, NPM: 4, 4; NCL: 3, 4) and zQ175 (N: number of mice, NPM: 5, 5; NCL: 3, 6) mice shows no significant differences. **C** Quantification of the mean area of the NPM1 or NCL signal (in μm^2^) per DAPI positive nuclei at 5 and 10 months (mo) in control (N for NPM1: 5, 4; for NCL: 3, 4) and zQ175 (N for NPM1: 4, 6; for NCL: 3, 5) mice shows a significant reduction in nucleolar NPM1 signal at 10 months by Mann–Whitney U test (*p* = 0.038). Error bars represent SEM. *p < 0.05.

To explore the hypothesis that perturbed nucleolar homeostasis detected in the striatum by NPM1 nucleolar mislocalization represents a histopathological marker of HD progression, we analyzed skeletal muscle (quadriceps) of controls and zQ175 mice at 5 and 10 months (Fig. 5A–C). At 5 months the number of DAPI positive nuclei showing either NPM1 or NCL signal was similar between zQ175 and control mice (Fig. 5B). The area of the nuclei was also similar between control and zQ175 mutant mice at 5 and 10 months (Fig. S7A, B). However, in 10-month-old zQ175 mice, the nuclear area displaying NPM1 immunosignal was about 30% decreased (Fig. 5C). Importantly, this reduction was specific, as it was not observed for NCL (Fig. 5C). These findings suggest a nucleolar phenotype in skeletal muscle of zQ175 mice at 10 months. As a read-out for altered nucleolar function, we analyzed if rRNA synthesis was altered in skeletal muscle of zQ175 mice. We studied pre-rRNA transcription and mature 18 S rRNA by qRT-PCR, and we detected at 10 months a significant (~50%) decrease of 47 S pre-rRNA in skeletal muscle of the zQ175 mice, in line with altered nucleolar function (Fig. S6H–J).

Next, we asked whether the changes in NPM1 signal in skeletal muscle of zQ175 mice at 10 months were secondary due to a compromised function of striatal neurons, or whether they were associated with mHTT expression in the skeletal muscle. To this end, we analyzed quadriceps from the TIF-IA^D1Cre^ mouse model, characterized by massive death of *D1r*-expressing MSNs at 3 months [26]. In this mHTT-independent model of striatal neurodegeneration, nuclear NPM1 signals were not reduced but rather significantly elevated in skeletal muscle, compared with control mice. These data suggest that the reduced NPM1 signal observed in the skeletal muscle of the zQ175 mice is linked to the peripheral effects of mHTT rather than to the degeneration of striatal neurons (Fig. S8).

### Reduced nucleolar localization of NPM1 in skeletal muscle biopsies of HD patients

**Fig. 6:**
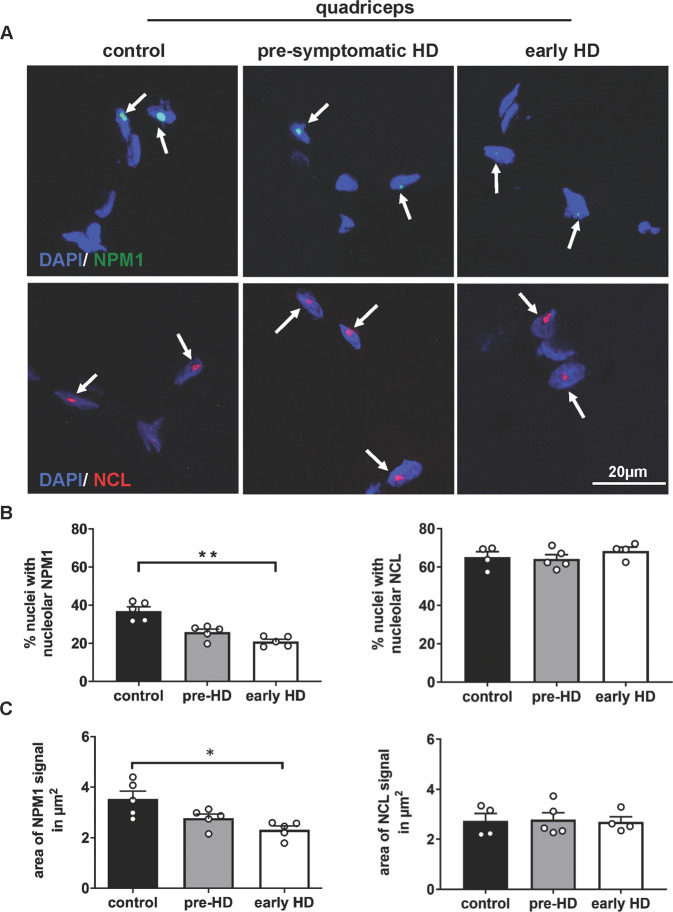
NPM1 signal is reduced in muscle (quadriceps) biopsies of early-Huntington’s disease patients. **A** Representative confocal images of human skeletal muscle (quadriceps) cryosections immunostained for NPM1 (green) or NCL (red) in HD patients at different stages and in age-matched controls. Nuclei are labeled with DAPI (blue). The arrows point to NPM1 or NCL signals. Scale bar: 20μm. **B** Quantification of the percentage of nuclei showing nucleolar NPM1 signal in HD patients in comparison with age-matched controls (N = 5 for each group), and indicating significant decreased NPM1 in early-HD by Kruskal–Wallis test and Dunn’s multiple comparison (*p* = 0.003 early-HD vs. controls). No significant differences in the percentage of nuclei with nucleolar NCL. **C** Mean area of the NPM1 signal (in μm^2^) per DAPI positive nuclei in control, pre- and early-HD individuals (N = 5 for each group). Statistically significant decrease of NPM1 signal area in early-HD compared with controls by Kruskal–Wallis test and Dunn’s multiple comparison (*p* = 0.01). No significant differences in the mean area of the NCL signal (in µm^2^) between the different groups. Error bars represent SEM. *p < 0.05, **p < 0.01; detailed statistical information is included in the Supplementary Statistical Information file.

For the critical transition of our findings from HD mouse models to the human disease, we analyzed skeletal muscle biopsies from HD patients and control individuals. More precisely, we investigated the NPM1 and NCL fluorescence-signal patterns in quadriceps from biopsies of nonaffected controls, presymptomatic, and early-symptomatic HD patients, similar as in the zQ175 mice. (Fig. 6A–C and Table S1).

In early HD patients, the percentage of DAPI positive nuclei showing nucleolar NPM1, and also the area of the NPM1 signal were about 2- and 1.5-fold lower than those of controls, respectively (Fig. 6B, C). In the pre-HD cohort, we observed a nonsignificant trend for reduced signals. A similar analysis performed for NCL showed no differences in these parameters at any stage (Fig. 6B, C). Nuclear areas were similar in all groups (Fig. S7C).

The same images were reanalyzed with an optimized automated approach based on the semantic convolutional neural network learning, as indicated in the Supplementary methods. Manually derived and automatically determined data displayed a strong correlation (Fig. S9). This automated approach represents a prerequisite for using NPM1 distribution in skeletal muscle as a marker for HD progression, and it opens the possibility to apply this analysis on a large scale of samples by digital pathology.

## Discussion

Nucleolar stress is associated with mHTT expression, and it is implicated in the response to cellular stress and in protein quality control. In this study, we provided the first in vivo evidences that this mechanism exacerbates motor phenotypes in a preclinical mouse model of HD, and that it alters mHTT intranuclear distribution. Aberrant rRNA processing in a striatal-derived cell model that expresses human mHTT indicated a novel mHTT-associated intrinsic dysfunctional mechanism. As summarized in Table S3, the nucleolar localization of the protein NPM1 was specifically reduced in the skeletal muscles of HD mice and in biopsies from HD patients, suggesting a novel candidate histopathological biomarker for monitoring HD progression in a peripheral tissue.

These findings are particularly important because changes in NPM1 distribution appear to be required for maintaining mHTT in a disperse state in the nucleoplasm. NPM1 and mHTT are known to interact [54, 55]. NPM1 contains disordered and low-complexity sequences that may render this protein prone to interact with mHTT. Recent evidence showed that mHTT has distinct dynamic states in living cells, including fast diffusion rates, dynamic clustering and stable aggregation [56]. Studies in HEK293T cells transfected with artificial beta-sheet proteins, mimicking prefibrillar and fibrillar aggregate formation suggested that NPM1 might shield mHTT aggregate surface [57]. Our finding that the disruption of nucleolar integrity, and NPM1 release from the nucleolus, which promotes the loss of a diffuse mHTT state, reconciles with the role of nucleolar integrity in protein quality control and formation of protein aggregates [4, 33, 34].

Previous studies showed that NPM1 is transiently upregulated in striatal neurons of the R6/2 transgenic mice before worsening of motor endpoints [58], and that its nucleolar localization is partially lost in the same model at a presymptomatic stage [26]. Accordingly, the zQ175 mice show that NPM1 nucleolar localization is partially lost at a presymptomatic stage, further corroborating that changes in NPM1 subcellular distribution might be pathogenic. These findings are in agreement with the biphasic nature of nucleolar stress observed, for example, in amyotrophic lateral sclerosis. In this condition, nucleolar stress occurs before the typical TDP-43 (TAR DNA-binding protein 43) proteinopathy [5]. A shared role for nucleolar stress in progressive neurodegenerative disorders, characterized by accumulation of intranuclear inclusions awaits future investigations [4, 59–61].

In turn, mHTT interaction with NPM1 might exert toxicity by affecting the multiple functions of NPM1. NPM1 has been involved in induction of autophagy upon inhibition of RNA Pol I [62]. Future studies should address whether the delocalization of NPM1 results in a deregulation of the autophagic process.

On the other hand, other pathogenic effects can be hypothesized. NPM1 impairment leads to an accumulation of pre-rRNA and various processing intermediates, as shown by NPM1 depletion experiments in human cells [46]. While NPM1 knockdown is toxic, its nuclear overexpression protects against mHTT-induced death in cultured cerebellar granule and cortical neurons [58]. We showed that the expression of human mHTT in striatal-derived cells is associated with qualitative alterations of ribosome production, namely the activation of cryptic processing sites, and that NPM1 loses its nucleolar distribution in the same model. At this stage, we cannot rule out that altered NPM1 affects pre-rRNA processing or sorting, as reported for another nucleolar protein fibrillarin in human cell cultures [63]. Future studies should address whether such changes in the kinetics of RNA cleavage impact other facets of ribosome biogenesis, such as rRNA modifications or pre-ribosome assembly, and result in the production of ribosomes with altered translational abilities. Interestingly, in a genome-wide screen in yeast, expression of a mHTT fragment (Htt103Q) causes a dramatic reduction in expression of genes involved in rRNA metabolism and ribosome biogenesis [64]. Moreover, cells expressing HttQ138 show a decreased translation by the prion-like protein and translation regulator Orb2, sequestered by mHTT aggregates [65]. Recently, mHTT has been reported to suppress protein synthesis in the same HD cell model adopted here by a mechanism involving ribosome stalling [66]. However, increased translation has been reported in a different progressive transgenic model of HD [67]. The phosphorylation of the eukaryotic translation initiation factor 4E (eIF4E) binding protein (4E-BP), an inhibitor of translation, increases in the striatum of R6/2 mice at a late manifest stage, supporting a time- and mRNA-specific dysregulation of translation leading to neurotoxicity, e.g., due to increased ribosomal protein translation [67–69].

Our findings point not only to nucleolar stress as a contributor to HD, but also to redistribution of nucleolar NPM1 as a cellular marker for HD progression. As summarized in Table S3, there are tissue-specific differences within the same model (striatum vs. skeletal muscle), model-specific differences within the striatum (R6/2 vs. zQ175 mice) and age-specific differences within the muscle (zQ175 at 5 and 10 months) for nucleolar transcription and integrity. It remains to be analyzed whether these differences in nucleolar function and integrity correlate with different levels and / or state of mHTT (fibrils vs. inclusion bodies) and/or NPM1. These studies will be also important for the validation of NPM1 as a histopathological biomarker in longitudinal studies and for testing beneficial and adverse effects of therapeutic intervention on HD progression. However, for clinical purposes needle biopsies might be better suitable being less invasive and implying a faster recovery than open biopsies [70]. In cancer research, changes in nucleolar size and shape are considered a reliable parameter to predict tumor growth and nucleolar proteins are evaluated as therapeutic targets [71, 72]. However, the value of the nucleolus as a potential biomarker is still neglected in the field of neurodegenerative diseases. We showed here, as a proof of principle, how the analysis of NPM1 could be automatized for clinical neuropathology applications, paving the way for high-throughput systematic analysis.

In conclusion, we identified nucleolar stress as a disease mechanism contributing to HD pathogenesis, and in particular NPM1 distribution pattern in skeletal muscle as a novel promising candidate for developing an accessible and reliable biomarker for HD progression, and possibly a target for therapeutics.

## Supplementary information

Suppl. Figure Legends and References

Suppl.Figures

Suppl.Tables 1_3

Suppl. Statistical Information

Full gene list in Suppl Fig 5

Checklist

## Data Availability

The raw data that support the findings of this study are available under “Supplementary statistical information” and from the corresponding author upon reasonable request.
