# Supplementary material for: Nucleolar stress controls mutant Huntington toxicity and monitors Huntington’s disease progression
Source: Cell Death Dis. 2021 Dec 8;12(12):1139. doi: 10.1038/s41419-021-04432-x (PMC8655027; doi:10.1038/s41419-021-04432-x)
Supplement: Supplementary file 1 — Suppl. Figure Legends and References [file 41419_2021_4432_MOESM1_ESM.docx]

**Supplementary Information**

**Supplementary Materials and Methods**

**Polysome profiling**

5x10^6^ StHdh Q7/Q7 and Q111/Q111 cells were treated with cycloheximide (100 µg/ml) for 5 min and washed once in cold PBS/cycloheximide (100 µg/ml). The cells were lysed in 400µl lysis buffer (15 mM Tris–HCl, pH 7.4, 15 mM MgCl_2_, 300 mM NaCl, 1% Triton X-100, 100 μg/ml cycloheximide, 1X complete protease inhibitors (Roche Applied Science, Mannheim, Germany), 0.1% β-mercaptoethanol). As RNAse inhibitor we used RNasin (200U/ml) (Thermo Fisher Scientific, Waltham, USA). The lysates were centrifuged at 10,000 g for 10 min at 4 °C, and the supernatants were applied to linear 17.5–50% sucrose gradients in 20 mM Tris–HCl (pH 7.4), 5 mM MgCl_2_, and 150 mM NaCl. Centrifugation was carried out at 36,000 rpm for 2.5 h at 4°C in a Beckmann SW60 rotor. Gradients were eluted with an ISCO UA-6 gradient fractionator, and polysome profiles were recorded by continuously monitoring the absorbance at 254 nm. The fraction of ribosomes engaged in translation was calculated by dividing the area under the polysomal part of the curve by the total area.

**SUrface SEnsing of Translation (SUnSET) assay**

StHdh Q7/Q7 and Q111/Q111 cells (90% confluent grown in Petri dishes 6 cm diameter) The cells were incubated with 1 μg/ml of puromycin for 30 min and then analyzed to detect the puromycin-labeled peptides by immunoblotting. The puromycin concentration was determined by preliminary experiments of cell viability at 0.1-1.5 μg/ml. For immunoblotting total extracts were prepared by re-suspending cells in RIPA buffer (25 mM Tris-HCl pH 7.6, 150 mM NaCl, 5 mM EDTA (Ethylenediaminetetraacetic acid), 1% Triton, 1% sodium deoxycholate, 0.1% SDS), 1 mM PMSF (phenylmethylsulfonyl fluoride), 1 mM DTT (Dithiothreitol), 1X proteinase inhibitor (Roche complete, EDTA free). Protein quantification was performed by using the Thermo Scientific Pierce BCA protein Assay kit (detergent compatible formulation). The whole extracts (20 μg) were loaded on 10% SDS-PAGE gel and blotted on PVDF (Polyvinylidene fluoride) membrane (Biorad, Hercule, USA) with wet apparatus (Biorad), for 60 min at 400 mA. After blocking in 5% dry milk in TBS, the membrane was incubated overnight with the appropriate primary antibodies. The primary antibodies used were: anti-puromycin (Millipore MABE343, 1:1000) and anti-beta actin (sc-47778, Santa Cruz Biotechnology, 1:1000). The secondary antibody was anti-mouse HRP (horseradish peroxidase)-linked (NA931, Amersham ECL Mouse IgG, HRP, GE Healthcare Life Science, Chicago, USA) diluted 1:10000 in 5% dry milk in PBS, and incubated for 1h at room temperature. After ECL (Electrochemiluminescence) assay (Pierce ECL Western Blotting Substrate cat. 32106 Thermo Fisher Scientific), the membrane was incubated with the film (Kodak) for protein detection. As additional loading control, the same amount of total protein extracts were loaded on 10% SDS-PAGE and then stained with Coomassie Blue R-250 (Sigma, Munich, Germany). Protein expression was quantified by densitometric analysis with the ImageJ Software.

**RNA in situ hybridization for quantification of RNA foci**

Non-radioactive RNA *in situ* hybridization (ISH) was performed on four to eight paraffin sections per mouse using a specific riboprobe hybridizing to regions in the leader sequence of the pre-rRNA as previously described (1). In brief after paraffin removal, sections were rehydrated and treated with proteinase K (10 μg/ml in 20 mM Tris/HCl, 1 mM EDTA, pH 7.2) for 7 min. Sections were incubated for 30 min in 2X SSC and in Tris/glycine buffer for at least 30 min until application of the hybridization mixture. The hybridization mixture was prepared as follows: 40% deionized formamide (Life Technologies, Calrsbad, USA), 5X SSC, 1X Denhardt's solution (ThermoFisher Scientific), 100 mg/ml salmon sperm DNA, 100 mg/ml yeast tRNA, 50 – 100 ng riboprobe, diluted in DEPC-H_2_O to a final volume of 100 µl per slide. Slides were incubated at 55 °C overnight. Post-hybridization steps were performed by washing the slides in 0.5X SSC/20% formamide at 60 °C and in NTE (0.5 M NaCl, 10 mM Tris pH 7.0, 5 mM EDTA) at 37 °C. Slides were then incubated with 10 µg/ml RNase A in NTE for 30 min at 37 °C. Samples were washed in pre-warmed 0.5X SSC/20% formamide for 30 min at 60 °C and kept in 2X SSC for 30 min at room temperature. Sections were then placed in blocking solution (1 % blocking reagent, 11096176001, Roche, diluted in 100 mM maleic acid, 150 mM NaCl, pH 7.5) for 10 min at room temperature. Subsequently, sections were incubated with alkaline phosphatase-conjugated anti-digoxygenin antibody overnight at 4 °C. NBT/BCIP liquid substrate system for alkaline phosphatase (11681451001, Roche) was used for the staining reaction at room temperature in the dark. Incubation was stopped after 4 – 5 h. Sections were counterstained for 10 min with Nuclear Fast Red (Vector Laboratories, Burlingame, USA), and subsequently the coverslip was mounted with Kaiser's Glycerol Gelatin (Merck, Darmstadt, Germany). In situ hybridization and immunohistochemistry images were acquired by a Leica DM6B microscope with a DMC2900 camera using a 20X objective.

For the quantification of the number of pre-rRNA foci by in situ hybridization the signals produced by labeling the riboprobe with digoxigenin were counted and normalized to the number of nuclei identified by counterstaining with the Nuclear Fast Red, as previously reported (2).

**Northern blot for 5.8S rRNA analysis**

RNA was extracted from the entire striatum of one mouse brain hemisphere using the Trizol method (ThermoFisher Scientific). 1-3 µg were separated using 15% urea-PAGE (ThermoFisher Scientific). Gels were stained with SYBR Gold (ThermoFisher Scientific), and immediately imaged and transferred to Nytran SuperCharge membranes (Schleicher and Schuell, New Hempshire, USA), and UV crosslinked with an energy of 0.12 J. Membranes were hybridized in 5X SSC, 20 mM Na_2_HPO_4_, pH7.4, 7% SDS, 1X Denhardt’s for at least 1 h at 42°C. For detection of 5.8S and 5S rRNA, membranes were incubated overnight (12–16 h) at 42°C with ^32^P-end-labelled oligonucleotides probes added in hybridization buffer with following sequence: 5.8S: 5’-GATGATCAATGTGTCCTGCAAT-3′; 5S: 5’-GGGTGGTATGGCCGTAGAC-3’. Following overnight incubation, the membranes were washed for 15 min at 43°C with 3X SSC, 5% SDS and for 15 min at room temperature with washing buffer (1X SSC, 1% SDS). Membranes were exposed on film, then stripped and re-hybridized with 5S probe for further analysis. Quantification of Northern blot signals from three independent experiments was performed on autoradiographs using Fiji/ImageJ (3).

**Behavioral tests**

Mice were tested for motor function on a rotarod apparatus (Ugo Basile, Gemonio, Italy) using a gradual acceleration protocol (4–40 rpm) up to maximal 8 min. Forelimb strength of all experimental groups was tested using a grip strength meter (Ugo Basile, model 47106), which automatically measured the force needed for the mouse to release its grip (4).

Rotarod and grip strength tests were performed 2 days after adapting to the respective tests at 9 weeks (trial 1), at 10 weeks (trial 2) and at 11 weeks (trial 3) for the experimental groups including control, TIF-IA^D1Cre^, R6/2 and their double mutant mice, and on the next day for the experimental groups including control and zQ175 mice (5 month-old). On the day of each trial each mouse was tested three times in intervals of 15 min. The mean endurance for the three runs (or recorded force, in millinewton) per mouse was calculated. The tests were performed blind for genotype.

**Immunofluorescence / Immunohistochemistry**

For immunofluorescence and immunohistochemistry, one brain hemisphere was fixed in 4% paraformaldehyde (PFA) in phosphate-buffered saline (PBS), pH7.4 overnight at 4 °C and paraffin embedded. Coronal sections (7 μm) were cut on a microtome (Leica 2235). The region of the striatum comprised between Bregma +0.74 mm and -0.34 mm was used for the histological analyses. Mouse skeletal muscle (quadriceps) was mounted in Tissue Freezing Medium (12020108926, Leica) and then snap-frozen in liquid N_2_-cooled 2-methylbutane and stored at -80°C until sectioning and immunostaining, as mentioned above.

Sections were cut in transverse orientation to the fiber direction at 12 µm using a Leica CM1859 cryostat. Cryosections were fixed in 4% PFA for 15 min before staining. For antigen retrieval the slides were boiled in 1X citrate buffer (HK086-9K, Biogenex, San Ramon, USA). Sections were blocked with 5% normal swine serum (NSS, S-4000, Vector Laboratories). Primary antibodies were diluted in 5% NSS and incubated over night at 4°C in a humidified chamber.

For immunohistochemistry, visualization of antigen-bound primary antibodies was carried out using a biotinylated secondary antibody together with the avidin-biotin system and the VECTOR peroxidase kit (PK-6100, Vector Laboratories) using both diaminobenzidine tablets (D4293, Sigma) as a substrate. For double immunofluorescence staining, slides were incubated with a second primary antibody as described before. Nuclei were stained with DAPI (4′,6-diamidino-2-phenylindole) (1:10^6^, 62248, ThermoFisher Scientific). Slides were mounted using AquaPolymount (18606-20, Polysciences, Niles, USA).

**Antibodies**

Antibodies for immunostaining were: anti-NCL (1:500, ab70493, abcam, Cambridge, UK) (2, 5), anti-NPM1/B23 (1:100, MAB4500, Millipore, Burlington, USA) (6, 7), sequestosome 1 (p62/SQSTM1) (1:100, P0067, Sigma), EM48 (1:100, MAB5374, Millipore) (8), S830 (1:1000; Neueder/Bates) (9). For confocal microscopy, secondary antibodies Alexa 488 (A-21206, 1:100, Thermo Scientific) and Alexa 594 (A-21207, 1:100, Thermo Scientific) were used. For stimulated emission depletion (STED) microscopy, Alexa 594 (A-21207, 1:100 Thermo Scientific) and Star Red (STAR-RED-1056, 1:100 Abberior GmbH) were used.

**Image analysis**

The percentage of nuclei showing NCL and NPM1 in the nucleoli was determined by counting the number of DAPI positive nuclei showing a circular nucleolar signal. The mean area of the nucleolar marker signal was determined by circling the signal area in each DAPI positive nucleus, considering in total only the double-labelled nuclei (10). The percentage of DAPI positive nuclei showing intranuclear mHTT inclusions and their mean area was measured in a similar way.

For the semi-quantitative analysis of the signal mean intensities, nuclei labelled with DAPI were identified and marked in maximal projection images and the DAPI negative region outside was considered as the background. A cut-off of 50 µm^2^ was applied to exclude smaller fragmented DAPI positive signals. The DAPI positive area identified the region of interest (ROI). The mean intensities of NCL in the entire nucleus and the respective background were measured in each of the 10 planes of the z-stack, and the intensities of every plane were summed up. The intensity of the signal in the ROI was subtracted by the background intensity for each nucleus. The relative mHTT subcellular distribution was defined by the ratio between mHTT mean intensities in and outside the nucleus (11). The intranuclear distribution was defined as the ratio between mHTT intensity in the nucleoplasm and in the inclusions (12). The region of the nucleus not occupied by the mHTT inclusions was considered as the nucleoplasm. For this analysis the mHTT inclusions were marked on the maximal z-projection. Intensities were measured across the whole z-stack and summed up. For each nucleus the intranuclear mHTT distribution was calculated by dividing the mHTT intensity in the nucleoplasm by the mHTT intensity in the inclusion.

Line profiles were measured after acquisition using ImageJ. A line through the nucleolus was drawn and measured using the Plot Profile function, as previously described (13-15).

The manual segmentation of NPM1 positive nucleoli in human skeletal muscle biopsies was automated by a semantic image segmentation algorithm based on deep learning. The neural network used here is a fully convolutional neural network (FCN) using exclusively convolutional operations and it can thus be applied to images of arbitrary size in a computationally efficient way (16). The detailed network architecture used here was developed and provided by Wolution GmbH & Co. KG, through its scientific image analysis platform, and it uses five convolutional layers as well as activation functions of ReLu type. The power of such architectures for medical applications was previously demonstrated (17). The results of the percentage of NPM1 positive nuclei and of the NPM1 signal area achieved by the manual and automated approach were then correlated, as indicated in the Supplementary Materials and Method section.

**Statistics**

Statistical analysis was performed with Graphpad Prism 7.04 software. Mean values per sample were used for statistical analysis. Datasets were analyzed for their statistical significance using non-parametric unpaired, two-tailed Mann-Whitney U (MWU) test or using the Kruskal-Wallis test followed by Dunn’s post-hoc analysis for multiple comparisons (18). Behavioral data were analyzed by two-way ANOVA with Tukey’s post-hoc analysis. For all tests statistical significance level was set at p<0.05. Complete details about statistical analysis are provided as **Supplementary Statistical Information**.

**Supplementary Figure Legends**

**Supplementary Figure 1: Expression of mutant Huntingtin in cells is associated with translational deficits.** (A) Representative polysome profiles of the Q7/7 and Q111/111 cells. (B) Ratio of polysomes to total ribosomes. Statistical significance by Mann-Whitney U test, * p<0.05. (C) Representative Western blot showing newly translated proteins in the Q7/7 and Q111/111 cells treated with puromycin (+) or untreated (-), the levels of β-actin and Coomassie staining as loading controls. (D) Translation rate determined by relative puromycin incorporation to β-actin. Statistical significance by Mann-Whitney U test, ** p<0.01. Values represent mean ±SEM.

**Supplementary Figure 2: Interaction between nucleolar stress and mHTT accumulation.** (A) Quantification of the percentage of nuclei with nucleolar NPM1 and NCL localization analysed in the four experimental groups as mean values. (N, number of mice; control: 4, TIF-IA^D1RCre^: 3, R6/2: 4, dm: 5). Statistical significance evaluated by Kruskal-Wallis test (p=0.0005) and post-hoc Dunn’s multiple comparison; * p<0.05, ** p<0.01 (% nuclei with nucleolar NCL: controls vs. TIF-IA^D1Cre^, p=0.008; controls vs. dm, p=0.015; TIF-IA^D1Cre^ vs. R6/2, p=0.017; R6/2 vs. dm, p=0.033. Percentage of nuclei with nucleolar NPM1: controls vs. TIF-IA^D1Cre^, p=0.033; controls vs. dm, p=0.037; TIF-IA^D1Cre^ vs. R6/2, p=0.22; R6/2 vs. dm, p=0.30). (B) Quantification of the percentage of striatal nuclei with intranuclear mHTT inclusions and of their area in R6/2 and dm mice at 9 weeks. Diagrams show mean values error bars represent SEM (N, number of mice; R6/2: 4, dm: 4). (C, D) Representative images of immunohistochemistry performed on striatal sections by the EM48 antibody. (E) Nuclear distribution of mHTT measured as the percentage of nuclei with diffuse mHTT per field of view (FOV). Diagrams show mean±SEM (N, number of mice; R6/2: 4, dm: 4). Statistical significance by Mann-Whitney U test (p=0.03). Arrows point to nuclei in insets as examples of nuclei with diffuse mHTT, while circled area shows an example of nuclei without mHTT in the nucleoplasm. Scale bar 100μm, inset 20μm.

**Supplementary Figure 3: Motor behaviour deficits in R6/2 mice are enhanced upon induction of nucleolar stress.** (A) Scheme of the time-points included in the behavioural analysis. (B) Number of clasping episodes in 30 s expressed as mean values ±SEM for controls, TIF-IA^D1Cre^, R6/2 and dm at 9 weeks (N, number of mice; control: 8, TIF-IA^D1RCre^: 7, R6/2: 11, dm: 10). The dm mice show statistically significant higher number of clasping episodes in comparison to controls (p=0.002) and TIF-IA^D1Cre^ mice (p= 0.0023) by Dunn’s multiple comparison after Kruskal-Wallis test (p= 0.0006). Legend: * p<0.05, ** p<0.01, *** p<0.001. (C) Body weight in control, TIF-IA^D1RCre^, R6/2 and dm mice expressed as expressed as mean values ±SEM. (D) Diagram showing endurance (in s) on an accelerating rotarod in the four experimental groups at different ages (9, 10 and 11 weeks, w). Significant variation between genotypes by two-way ANOVA (p<0.0001); significant differences between groups for each age were determined by Tukey’s multiple comparison: R6/2 vs. dm: p=0.012 at 10 weeks, p=0.003 at 11 weeks (see complete information in the Supplementary Statistical Table). N: number of mice, control: 8, TIF-IA^D1RCre^: 6, R6: 12, dm: 12. (E) Diagram showing the results of grip strength test in the four experimental groups at different ages (9, 10 and 11 weeks, w). Significant variation in age and genotype by two-way ANOVA (p< 0.0006); significant differences between groups for each age were determined by Tukey’s multiple comparison. N control: 8, TIF-IA^D1RCre^: 6, R6: 12, dm: 12. Legend: * p<0.05, ** p<0.01, *** p<0.001; detailed statistical information is included in the Supplementary Statistical Information file.

**Supplementary Figure 4: Motor behavior analysis of pre-symptomatic zQ175 mice at 5 months.** (A) Body weight of zQ175 and control mice at 5 months shows no significant differences between control and mutant. (B) Endurance on an accelerating rotarod shows no significant differences at 5 months between control and zQ175 mice by Mann-Whitney U test (N: number of mice, control = 10, zQ175= 13). (C) Grip strength test at 5 months shows no differences between control and zQ175 mice. N: number of mice (in blue: males, in red: females), control = 10, zQ175= 13; values represent mean ±SEM.

**Supplementary Figure 5: Dysregulation analysis of rRNA transcription and processing factors in the zQ175 mice.** (A, B) The log2 fold changes of rRNA transcription and rRNA processing factors are shown for the analysis of human BA4 cortex RNAseq data (GSE79666)(19) and data from the striata and gastrocnemius of zQ175 mice at different ages (HDinHD.org)(20). rRNA transcription factors were chosen according to (21, 22) and rRNA processing factors according to (23). Only genes that were significantly changed (Benjamini-Hochberg adjusted *p*-value < 0.05) in at least one of the analysis datasets are shown. For the full list see Excel file.

**Supplementary Figure 6: rDNA transcription is unaffected in striata of zQ175 mice at both pre- and early manifest stages and it is downregulated in the muscle at an early manifest stage.** (A) Schematic representation of the 47S pre-rRNA transcript including the position of the primers used for qRT-PCR and of the riboprobe used for RNA ISH within the 5’-external transcribed spacer (5’-ETS). (B-D) Analysis of 47S pre-rRNA and mature 18S rRNA relative expression in the striatum by qRT-PCR at 5 and 10 months in controls (N= 6-8) and zQ175 (N= 4-6) shows no significant differences by Mann-Whitney U test. (E) Representative RNA ISH images of 5 and 10 month-old control and zQ175 mice showing the 47S pre-rRNA signals as blue punctuate signals within the nuclei in striatal sections. Scale bar: 20μm. (F) No significant difference in the average number of 47S pre-rRNA foci per striatal cell at 5 and 10 months in control (N= 9,7) and zQ175 mice (N= 7,6) by Mann-Whitney U test (p=0.09 at 5 months). (G) Representative Northern blots for the analysis of 5.8S rRNA in striata from controls (N= 10) and zQ175 (N= 8) at 5 months and densitometric analysis normalized by 5S rRNA. (H-J) Analysis of 47S pre-rRNA and mature 18S rRNA levels by qRT-PCR at 5 and 10 months in the skeletal muscle of controls (N= 3-4) and zQ175 (N= 5-7) expressed as fold change to respective controls. 47S pre-rRNA is significantly reduced using primer pair 47S2 on the zQ175 mice at 10 months by Mann-Whitney U test (p= 0.012). Values represent mean ±SEM. * p<0.05.

**Supplementary Figure 7: No differences in the nuclear area in the skeletal muscle of zQ175 mice and Huntington’s disease patients.** (A, B) Mean area of DAPI stained nuclei at 5 and 10 months in muscle (quadriceps) of control (N=5, 4) and zQ175 (N=4, 6) mice. (C) No significant differences between the three groups (N= 5 for each group) in the nuclear area assessed by DAPI signal area. Values represent mean ±SEM.

**Supplementary Figure 8: NPM1 signal area is not affected in the skeletal muscle of mutant mice showing striatal neurodegeneration.** (A) Representative confocal images of quadriceps cryosections stained for NPM1 (green) in control and TIF-IA^D1Cre^ mice at 3 months. Nuclei are labelled with DAPI (blue). The arrows point out to NPM1 signal. Scale bar: 20μm. (B) Quantification of the percentage of nuclei with nucleolar localisation of NPM1 in control (N=6) and TIF-IA^D1Cre^ mice (N= 4); p=0.0095 by Mann-Whitney U test. Mean area of the NPM1 signal (in μm^2^) in control (N=5) and TIF-IA^D1Cre^ (N= 4); p= 0.0317 by Mann-Whitney U test. Error bars represent SEM. * p<0.05, ** p<0.01.

**Supplementary Figure 9: Correlation between manual and automated counting of NPM1 signals and area in human muscle biopsies from healthy controls and pre-/early Huntington’s disease.** (A,B) Representative original and segmented images are shown for comparison. Scale bar: 10μm. (C) Quantification of the percentage of nuclei showing nucleolar NPM1 signal by a machine learning algorithm (semantic convoluted neuronal network) in pre- and early-Huntington’s disease individuals in comparison with age-matched controls (N=5 for each group). A significant decrease of NPM1 signal was confirmed in early Huntington’s disease by Kruskal-Wallis test and Dunn’s multiple comparison (p=0.002 early-Huntington’s disease vs. controls). (D) Pearson correlation between the manual and automated quantification is significant (p<0.0001). (E) Mean area of the NPM1 signal (in μm^2^) in control, pre- and early- Huntington’s disease individuals (N=5 for each group). Statistical significance is assessed by Kruskal-Wallis test and Dunn’s multiple comparison (p=0.027 early Huntington’s disease vs. control). (F) Pearson correlation between the manual and automated quantification is significant (p=0.0003). * p<0.05, ** p<0.01.

**Supplementary References**

1. Rieker C, Engblom D, Kreiner G, Domanskyi A, Schober A, Stotz S, et al. Nucleolar disruption in dopaminergic neurons leads to oxidative damage and parkinsonism through repression of mammalian target of rapamycin signaling. J Neurosci. 2011;31(2):453-60.

2. Evsyukov V, Domanskyi A, Bierhoff H, Gispert S, Mustafa R, Schlaudraff F, et al. Genetic mutations linked to Parkinson's disease differentially control nucleolar activity in pre-symptomatic mouse models. Dis Model Mech. 2017;10(5):633-43.

3. Schindelin J, Arganda-Carreras I, Frise E, Kaynig V, Longair M, Pietzsch T, et al. Fiji: an open-source platform for biological-image analysis. Nat Methods. 2012;9(7):676-82.

4. Neureither F, Ziegler K, Pitzer C, Frings S, Mohrlen F. Impaired Motor Coordination and Learning in Mice Lacking Anoctamin 2 Calcium-Gated Chloride Channels. Cerebellum. 2017;16(5-6):929-37.

5. Potapova TA, Unruh JR, Yu Z, Rancati G, Li H, Stampfer MR, et al. Superresolution microscopy reveals linkages between ribosomal DNA on heterologous chromosomes. J Cell Biol. 2019;218(8):2492-513.

6. Wang HF, Takenaka K, Nakanishi A, Miki Y. BRCA2 and nucleophosmin coregulate centrosome amplification and form a complex with the Rho effector kinase ROCK2. Cancer Res. 2011;71(1):68-77.

7. Parlato R, Rieker C, Turiault M, Tronche F, Schutz G. Survival of DA neurons is independent of CREM upregulation in absence of CREB. Genesis. 2006;44(10):454-64.

8. Davies SW, Turmaine M, Cozens BA, DiFiglia M, Sharp AH, Ross CA, et al. Formation of neuronal intranuclear inclusions underlies the neurological dysfunction in mice transgenic for the HD mutation. Cell. 1997;90(3):537-48.

9. Moffitt H, McPhail GD, Woodman B, Hobbs C, Bates GP. Formation of polyglutamine inclusions in a wide range of non-CNS tissues in the HdhQ150 knock-in mouse model of Huntington's disease. PLoS One. 2009;4(11):e8025.

10. Tiku V, Kew C, Mehrotra P, Ganesan R, Robinson N, Antebi A. Nucleolar fibrillarin is an evolutionarily conserved regulator of bacterial pathogen resistance. Nat Commun. 2018;9(1):3607.

11. Gasset-Rosa F, Chillon-Marinas C, Goginashvili A, Atwal RS, Artates JW, Tabet R, et al. Polyglutamine-Expanded Huntingtin Exacerbates Age-Related Disruption of Nuclear Integrity and Nucleocytoplasmic Transport. Neuron. 2017;94(1):48-57 e4.

12. Frottin F, Schueder F, Tiwary S, Gupta R, Korner R, Schlichthaerle T, et al. The nucleolus functions as a phase-separated protein quality control compartment. Science. 2019;365(6451):342-7.

13. Riback JA, Zhu L, Ferrolino MC, Tolbert M, Mitrea DM, Sanders DW, et al. Composition-dependent thermodynamics of intracellular phase separation. Nature. 2020;581(7807):209-14.

14. Taslimi A, Vrana JD, Chen D, Borinskaya S, Mayer BJ, Kennedy MJ, et al. An optimized optogenetic clustering tool for probing protein interaction and function. Nat Commun. 2014;5:4925.

15. Zhu L, Richardson TM, Wacheul L, Wei MT, Feric M, Whitney G, et al. Controlling the material properties and rRNA processing function of the nucleolus using light. Proc Natl Acad Sci U S A. 2019;116(35):17330-5.

16. Shelhamer E, Long J, Darrell T. Fully Convolutional Networks for Semantic Segmentation. IEEE Trans Pattern Anal Mach Intell. 2017;39(4):640-51.

17. Havaei M, Davy A, Warde-Farley D, Biard A, Courville A, Bengio Y, et al. Brain tumor segmentation with Deep Neural Networks. Med Image Anal. 2017;35:18-31.

18. Altman DG, Gore SM, Gardner MJ, Pocock SJ. Statistical guidelines for contributors to medical journals. Br Med J (Clin Res Ed). 1983;286(6376):1489-93.

19. Lin L, Park JW, Ramachandran S, Zhang Y, Tseng YT, Shen S, et al. Transcriptome sequencing reveals aberrant alternative splicing in Huntington's disease. Hum Mol Genet. 2016;25(16):3454-66.

20. Langfelder P, Cantle JP, Chatzopoulou D, Wang N, Gao F, Al-Ramahi I, et al. Integrated genomics and proteomics define huntingtin CAG length-dependent networks in mice. Nat Neurosci. 2016;19(4):623-33.

21. Drygin D, Rice WG, Grummt I. The RNA polymerase I transcription machinery: an emerging target for the treatment of cancer. Annu Rev Pharmacol Toxicol. 2010;50:131-56.

22. Sharifi S, Bierhoff H. Regulation of RNA Polymerase I TranscriptioninDevelopment, Disease, and Aging. Annu Rev Biochem. 2018;87:51-73.

23. Aubert M, O'Donohue MF, Lebaron S, Gleizes PE. Pre-Ribosomal RNA Processing in Human Cells: From Mechanisms to Congenital Diseases. Biomolecules. 2018;8(4).
