# Supplementary material for: Nucleolar stress controls mutant Huntington toxicity and monitors Huntington’s disease progression
Source: Cell Death Dis. 2021 Dec 8;12(12):1139. doi: 10.1038/s41419-021-04432-x (PMC8655027; doi:10.1038/s41419-021-04432-x)
Supplement: Supplementary file 2 — Suppl.Figures [file 41419_2021_4432_MOESM2_ESM.pdf]

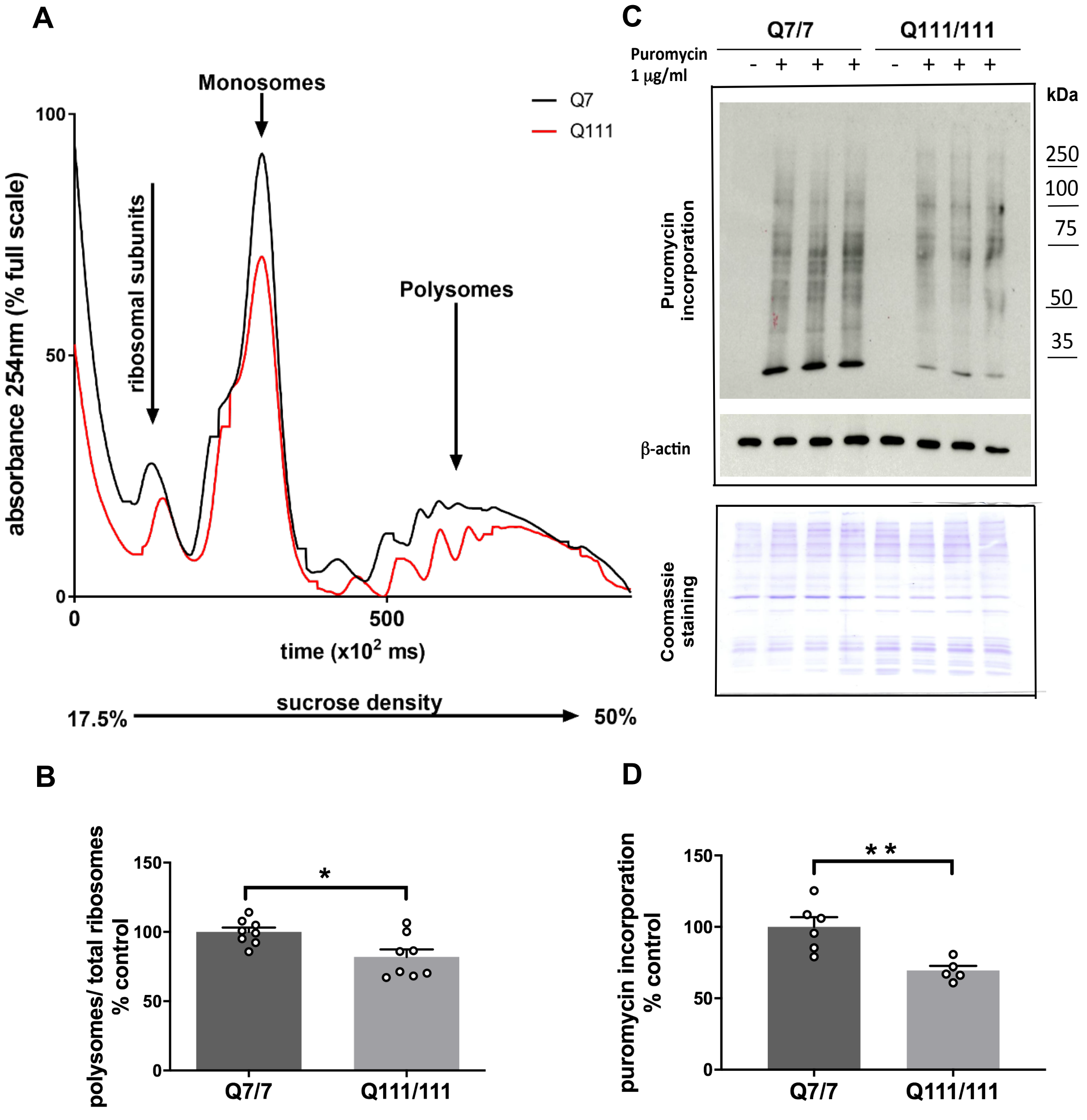

Suppl. Figure 1

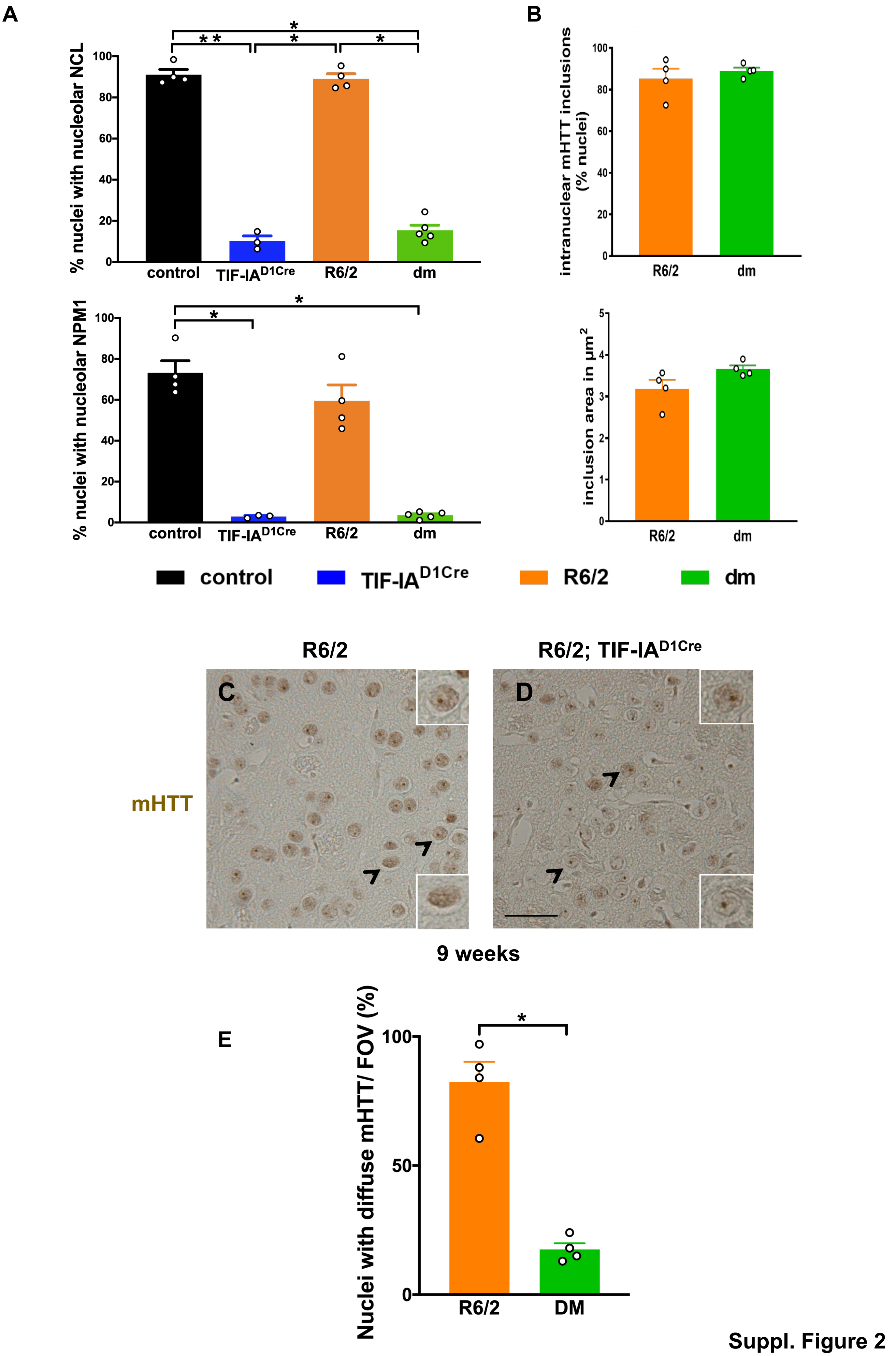

Suppl. Figure 2

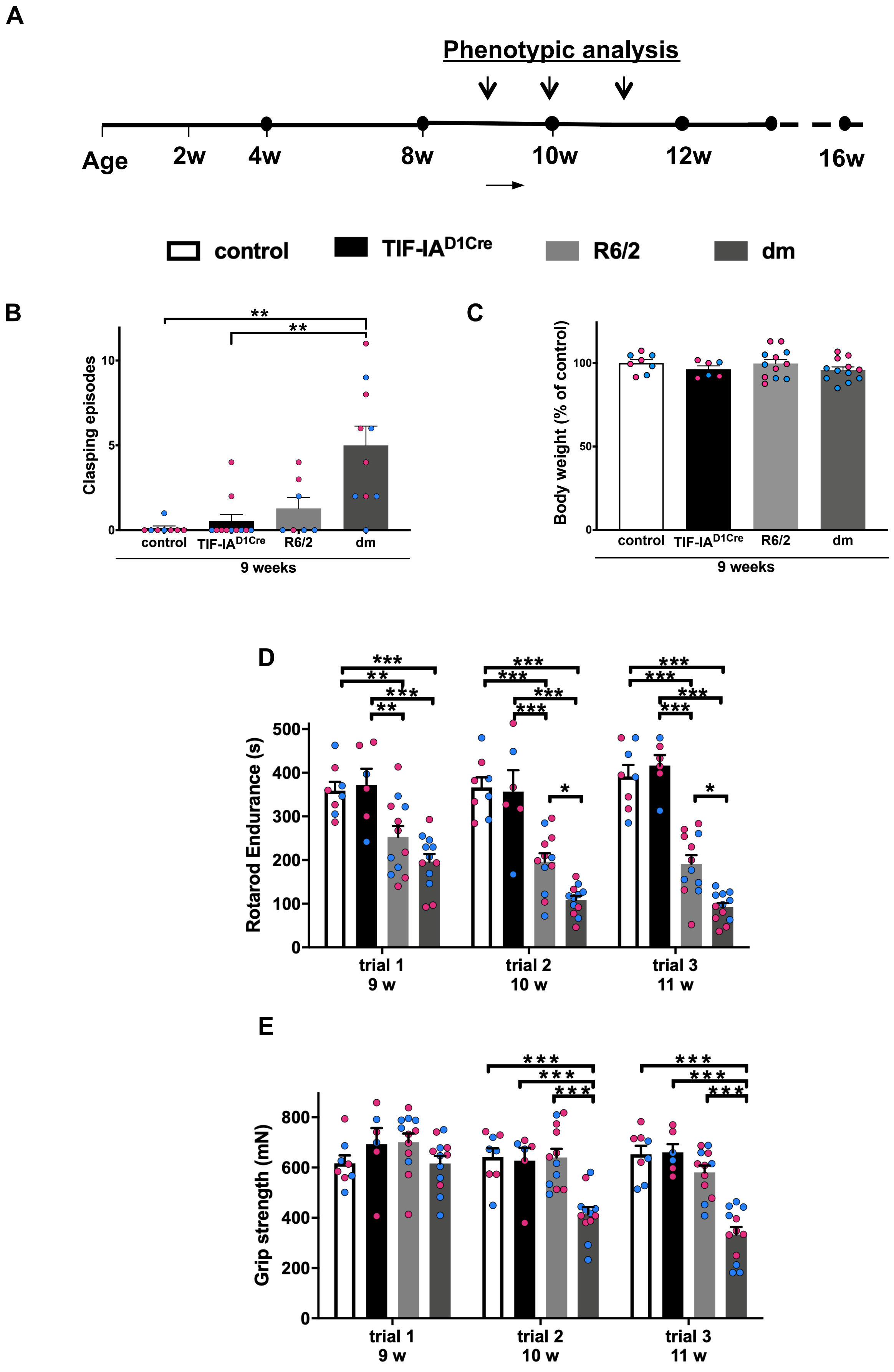

Suppl. Figure 3

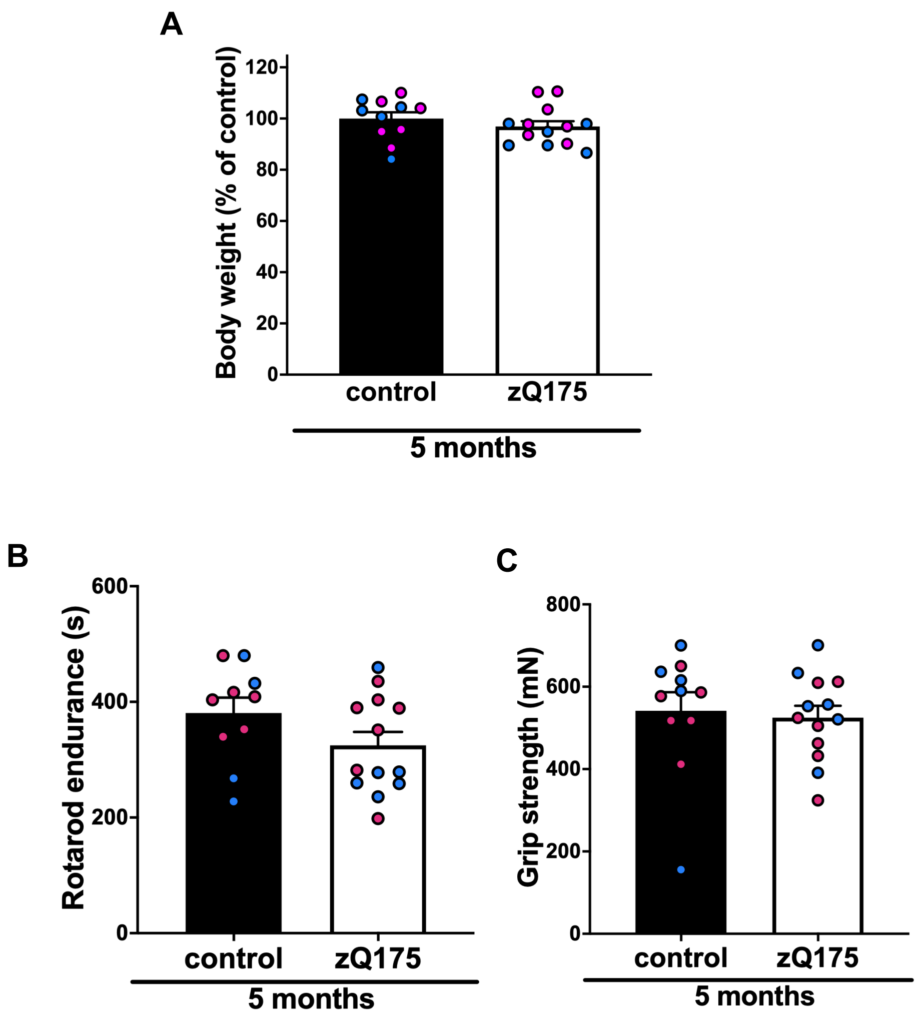

Suppl. Figure 4

A **rRNA transcription**

Gene	human BA4 cortex	zQ175 striatum 2 mth	zQ175 striatum 6 mth	zQ175 striatum 10 mth	zQ175 gastrocnemius 6 mth
RRN3	0.29313315				
UBTF		-0.1757289	-0.320152	-0.252921	
GTF2H2				-0.129181	
ERCC6			-0.1514219	-0.1519405	
TAF1C			-0.0894431		
GTF2H3			-0.0754702		
ERCC2			0.06336042		
PCNA			0.09297059		
KAT2B			0.10771751	0.12790748	
GTF2H5			0.10827059		
DDX21			0.11621982	0.18567179	
TOP1			0.11746182		
POLR1A			0.14489715		
ERCC3			0.17364773	0.23119398	
MYO1C			0.18749787	0.2273397	
POLR1E			0.21095236		
XRCC5			0.24006573	0.19054009	

B **pre-rRNA processing**

Gene	human BA4 cortex	zQ175 striatum 2 mth	zQ175 striatum 6 mth	zQ175 striatum 10 mth	zQ175 gastrocnemius 6 mth
NHP2			-0.3335672	-0.2729504	
RPL31			-0.2410299	-0.2047134	
LTN1			-0.0901754		
RPS19			-0.0821946		
SBDS			-0.0782517		
EXOSC10			-0.0749136		
RPS24			-0.0661457		
EFL1			0.08088634		
RPL18			0.09356379		
NPM1			0.09983137		
TCOF1			0.1492631		
DIS3			0.17113883	0.1884542	
FBL			0.22095978	0.20631843	

log2 fold change

< -1 0 > 1

Suppl. Figure 5

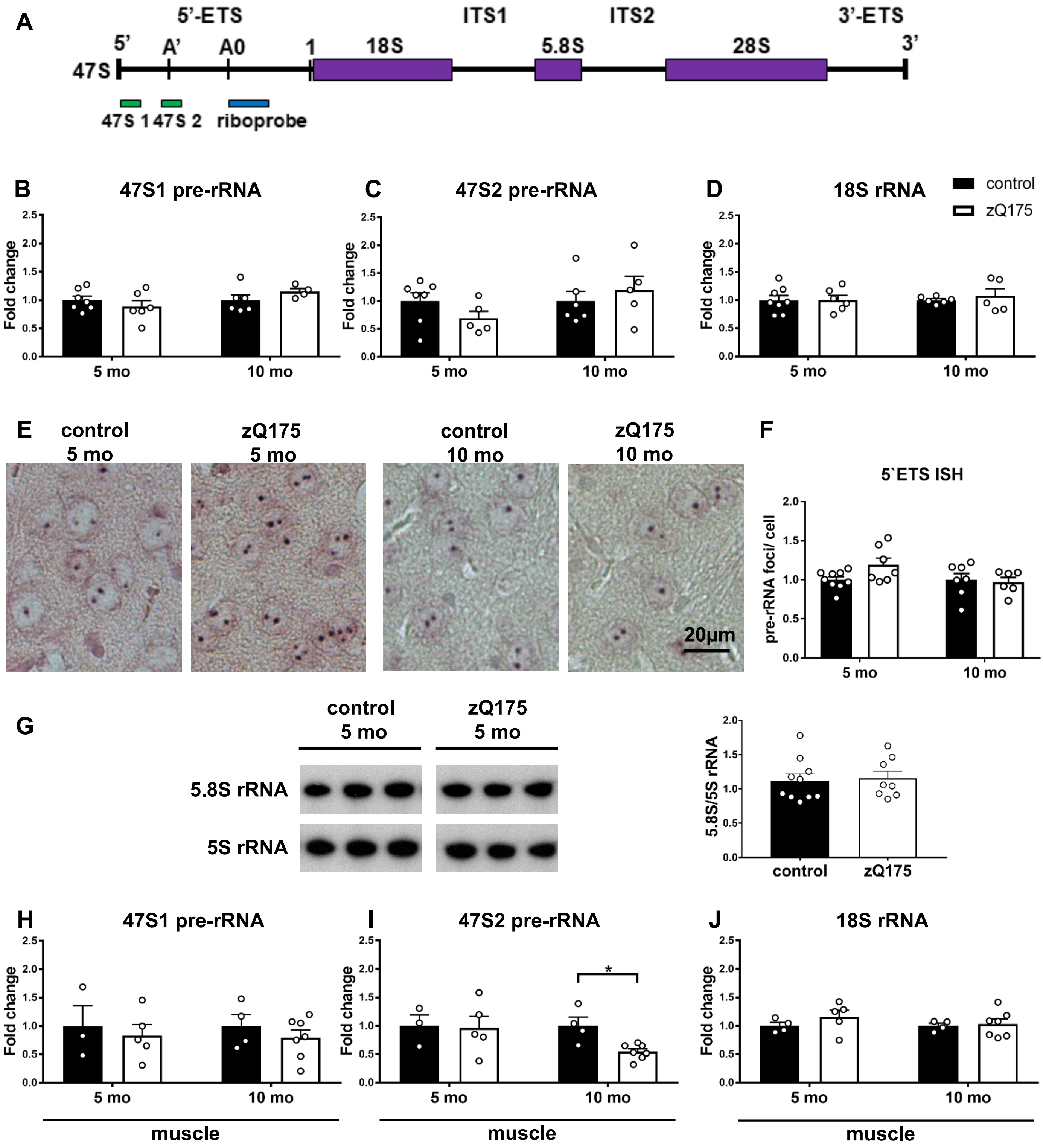

Suppl. Figure 6

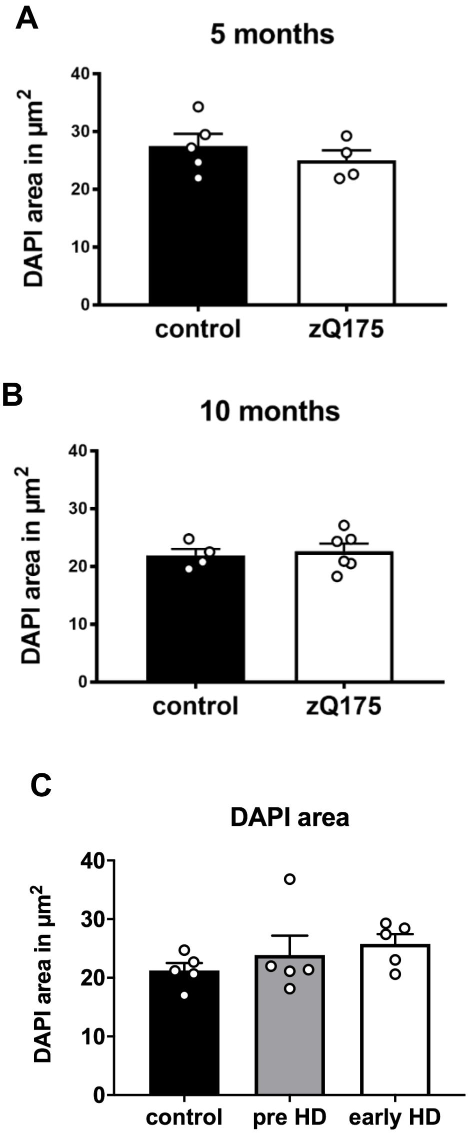

Suppl. Figure 7

A

B

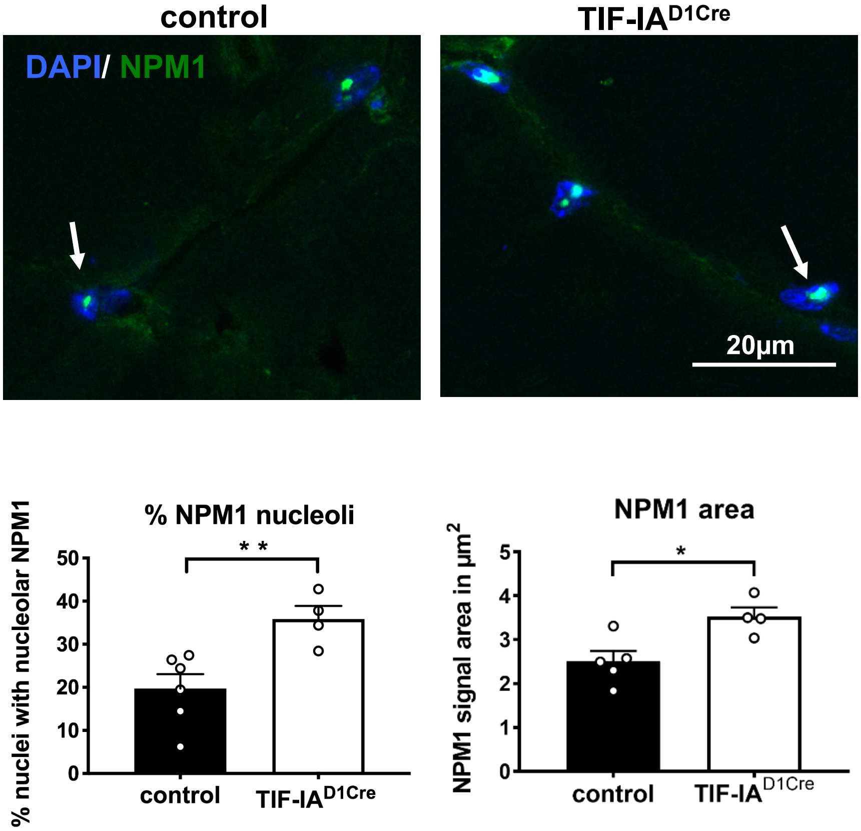

Suppl. Figure 8

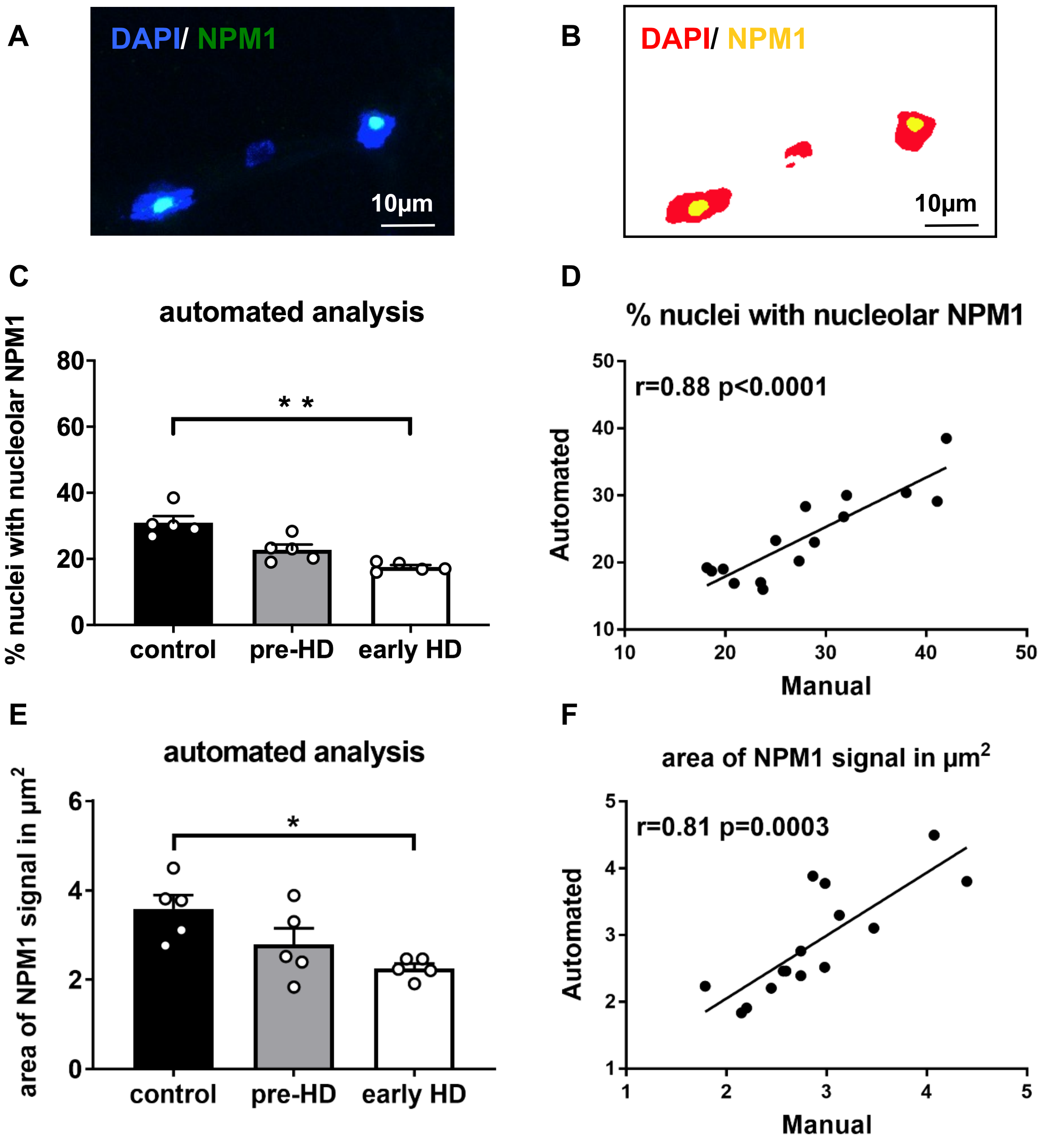

Suppl. Figure 9
