# Supplementary material for: Nucleolar stress controls mutant Huntington toxicity and monitors Huntington’s disease progression
Source: Cell Death Dis. 2021 Dec 8;12(12):1139. doi: 10.1038/s41419-021-04432-x (PMC8655027; doi:10.1038/s41419-021-04432-x)
Supplement: Supplementary file 3 — Suppl.Tables 1_3 [file 41419_2021_4432_MOESM3_ESM.docx]

**Supplementary Table 1: Summary of the human cohorts for which quadriceps biopsies**

**were analyzed.**

| **Patient cohort** | **control** | **pre-HD** | **early HD** |
| --- | --- | --- | --- |
| Age (years) | 38 ± 3.9 | 43.2 ± 9.1 | 43.4 ± 5.2 |
| Gender | 1 m / 4 f | 3 m /2 f | 2 m /3 f |
| Number of CAG repeats | n/a | 43.6 ± 1.5 | 45.2 ± 2.8 |
| DBS (=age*(CAG-35.5)) | n/a | 346 ± 73.4 | 420.5 ± 132.6 |

*Abbreviations in Table: m, male; f, female; DBS, disease burden score;* ± SD

**Supplementary Table 2: List of mouse TaqMan assays**

| **Gene** | **Assay ID** | **Primer/probe or consensus sequence** | **Gene –bank No.** | **Length**  **(bp)** | **EB** |
| --- | --- | --- | --- | --- | --- |
| *Rn18s* | Mm03928990_g1 | TACTTGGATAACTGTGGTAATTCTA | NR_003278.3 | 61 | - |
| *D1r* | Mm01353211_m1 | CCCAGATCGGGCATTTGGAGAGATG | NM_010076.3 | 65 | 1-2 |
| *D2r* | Mm00438541_m1 | GTCGTCTATCTGGAGGTGGTGGGTG | NM_010077.2 | 71 | 2-3 |
| *Hprt* | Mm01545399_m1 | GGACTGATTATGGACAGGACTGAAA | NM_013556.2 | 81 | 2-3 |
| *Metap1* | Mm00558361_m1 | ACTTCTGCTCGCAGGAATGCTTTAA | NM_175224.4 | 56 | 1-2 |

*Abbreviations: Length, amplicon length; EB: exon boundary*

**Supplementary Table 3: Overview of the functional and structural changes of the nucleolus by the analysis of in pre-rRNA synthesis and NPM1 and NCL immunostaining in different models of Huntington’s disease and in human muscle biopsies.**

|  | Q111/111  cells | R6/2  striatum  pre-/sym | zQ175 striatum  pre-sym | zQ175  quadriceps  sym | HD  quadriceps |
| --- | --- | --- | --- | --- | --- |
| Pre-rRNA | = ^a)^ | -^a) b)^ | = | - | -^c)^ |
| NPM1 in nucleoli | - | -^a)^ | - | - | - |
| NCL in  nucleoli | = | = | = | = | = |

***Abbreviations in Table:*** *pre-sym, pre-symptomatic; sym, symptomatic; HD, Huntington’s disease;*

*-, decreased; +, increased; ^a)^ Lee J et al, 2011; ^b)^ Kreiner et al. 2013; ^c)^ Jesse et al. 2017.*
