# Supplementary material for: Nucleolar stress controls mutant Huntington toxicity and monitors Huntington’s disease progression
Source: Cell Death Dis. 2021 Dec 8;12(12):1139. doi: 10.1038/s41419-021-04432-x (PMC8655027; doi:10.1038/s41419-021-04432-x)
Supplement: Supplementary file 4 — Suppl. Statistical Information [file 41419_2021_4432_MOESM4_ESM.pdf]

Supplementary statistical information

Table 1

Data and statistics for graphs shown in **Figure 1B**, n: number of nuclei, N: number of microscopic fields in two independent experiments. Statistical significance for NCL was assessed according to two-tailed t-test. Mann-Whitney U test (MWU) was not applicable due to lack of variance in the Q7/7 samples.

**B) % nuclei with nucleolar NPM1**						
	**mean**	**±SD**	**±SEM**	**median**	**95% CI**	**n/N**
**Q7/7**	82.75	12.87	5.255	82.35	69.24- 96.25	421/6
**Q111/111**	59.26	14.38	5.871	56.74	44.17-74.35	634/6
**MWU**	**0.0152**					
t-test	0.0138					

**B) % nuclei with nucleolar NCL**						
	**Mean**	**±SD**	**±SEM**	**median**	**95% CI**	**n/N**
**Q7/7**	100	0	0	100	100-100	180/11
**Q111/111**	95.45	7.778	3.175	98.21	87.29-103.6	135/6
**t-test**	0.0646					

Table 2

Data and statistics for graphs shown in **Suppl. Figure 1B, D**; N: total number of repeats performed respectively in four and two independent experiments. * Statistical significance is shown according to the Mann-Whitney U test (MWU).

**B) polysomes / total ribosomes (% of control)**						
	**Mean**	**±SD**	**±SEM**	**median**	**95% CI**	**N**
**Q7/7**	100	9.03	3.19	100	92.5-107.6	8
**Q111/111**	81.99	15.3	5.41	78.6	69.2-94.8	8
**MWU**	**0.038**					
**t-test**	0.012					

**D) puromycin incorporation (% of control)**						
	**Mean**	**±SD**	**±SEM**	**median**	**95% CI**	**N**
**Q7/7**	100	16.9	6.9	100.8	82.3-117.7	6
**Q111/111**	69.4	7.5	3.4	67.0	60.1-78.7	5
**MWU**	**0.0087**					
**t-test**	0.0047					

Table 3

Data and statistics for graphs shown in **Fig. 2B**, n: number of analysed striatal nuclei, N: number of mice. Statistical significance is shown according to the Mann-Whitney U test (MWU).

**B) mHTT intensity (nucleoplasm / inclusion ratio)**						
	**mean**	**±SD**	**±SEM**	**Median**	**95% CI**	**n/N**
**R6/2**	0.25	0.03	0.01	0.25	0.20-0.29	335/4
**dm**	0.18	0.01	0.01	0.18	0.16-0.20	296/4
***MWU**	**0.0286**					
**t-test**	0.0046					

Table 4

Data and statistics for graphs shown in **Suppl**. **Fig. 2**, n: number of analysed striatal nuclei, N: number of mice. Statistical significance is shown according to Kruskal-Wallis test (non-parametric one way analysis of variance).

**A) % nuclei with nucleolar NCL**						
	**Mean**	**±SD**	**±SEM**	**Median**	**95% CI**	**n/N**
**control**	91.09	5.01	2.50	89.31	83.12-99.06	217/4
**TIF-IA^{D1Cre}**	10.23	4.28	2.47	9.524	-0.39-20.85	185/3
**R6/2**	89.05	4.91	2.45	88.08	81.25-96.86	335/4
**dm**	15.36	5.67	2.53	13.68	8.322-22.4	371/5

**Kruskal-Wallis test p=0.0005**
**Dunn's multiple comparison**
**control vs. TIF-IA^{D1Cre} 0.0079, control vs. R6/2** 0.7664**, control vs. dm 0.0146, TIF-IA^{D1Cre} vs. R6/2 0.0172, TIF-IA^{D1Cre} vs. dm** 0.5914**, R6/2 vs. dm 0.0332**

**A) % nuclei with nucleolar NPM1**						
	**Mean**	**±SD**	**±SEM**	**Median**	**95% CI**	**n/N**
**control**	73.2	11.8	5.9	69.5	54.5-92	280/4
**TIF-IA^{D1Cre}**	2.9	0.7	0.4	3.2	1.1-4.7	241/3
**R6/2**	59.5	15.5	7.8	55.4	34.8-84.2	343/4
**dm**	3.6	1.6	0.7	3.9	1.5-5.6	464/5

**Kruskal-Wallis test p=0.0003**
**Dunn's multiple comparison**
**control vs. TIF-IA^{D1Cre} 0.0333, control vs. R6/2** >0.9999**, control vs. dm 0.0369, TIF-IA^{D1Cre} vs. R6/2** 0.2221**, TIF-IA^{D1Cre} vs. dm** >0.9999**, R6/2 vs. dm** 0.3021

**B) Intranuclear mHTT inclusions (% nuclei)**						
	**Mean**	**±SD**	**±SEM**	**Median**	**95% CI**	**n/N**
**R6/2**	85.20	9.44	4.72	87.03	70.18-100.2	335/4
**dm**	88.97	3.16	1.58	89.07	83.94-93.99	296/4
**MWU**	0.8857					
**t-test**	0.4781					

B) mHTT inclusion area (µm^2)						
	**Mean**	**±SD**	**±SEM**	**Median**	**95% CI**	**n/N**
**R6/2**	3.18	0.44	0.22	3.296	2.49- 3.88	335/4
**dm**	3.66	0.18	0.09	3.614	3.38-3.94	296/4
**MWU**	0.1143					
**t-test**	0.0894					

E) Nuclei with diffuse mHTT/field of view (FOV) (%)						
	**mean**	**±SD**	**±SEM**	**Median**	**95% CI**	**n/N**
**R6/2**	82.38	15.56	7.78	86.00	57.6- 107.1	575/4
**dm**	17.50	4.80	2.40	16.50	9.87-25.1	584/4
**MWU**	**0.0286**					
**t-test**	0.0002					

Table 5

Data and statistics for graphs shown in **Suppl. Fig. 3**, N: number of mice, w: weeks. * Statistical significance is shown according to Kruskal-Wallis test (non-parametric one way analysis of variance) or two-way ANOVA.

B) Clasping episodes							
	**mean**	**±SD**	**±SEM**	**Median**	**95% CI**	**N**	**m/f**
**Control**	0.13	0.35	0.13	0	-0.17-0.42	8	3/5
**TIF-IA^{D1Cre}**	0.55	1.29	0.39	0	-0.32-1.41	11	4/7
**R6/2**	1.29	1.70	0.64	0	-0.29-2.86	7	4/3
**dm**	5.00	3.59	1.14	5	2.43-7.57	10	5/5
***Kruskal-Wallis test p=0.0006**							
**p values from Dunn's multiple comparisons** Control vs. TIF-IA^{D1Cre} >0.9999, **Control vs. R6/2**>0.9999, **Control vs. dm 0.0017, TIF-IA^{D1Cre} vs. R6/2** >0.9999, **TIF-IA^{D1Cre} vs. dm 0.0023, R6/2 vs. dm** 0.1453							

C) Body weight (% of control)						
	**mean**	**±SD**	**±SEM**	**Median**	**95% CI**	**N (m/f)**
**Control**	100	5.7	2.0	100.6	95.2-104.8	8 (4/4)
**TIF-IA^{D1Cre}**	96.3	4.9	2.0	96.4	91.2-101.4	6 (2/4)
**R6/2**	99.7	8.7	2.5	99.3	94.2-105.2	12 (5/7)
**dm**	95.8	6.6	1.9	95.7	91.6-99.6	12 (7/5)
***Kruskal-Wallis test** p=0.424						
**p values from Dunn's multiple comparisons** **Control vs. TIF-IA^{D1Cre}** >0.9999, **Control vs. R6/2** >0.9999, **Control vs. dm** =0.734, **TIF-IA^{D1Cre} vs. R6/2** >0.9999, **TIF-IA^{D1Cre} vs. dm** >0.9999, **R6/2 vs. dm** >0.9999						

D) Rotarod endurance (s)				
		trial 1<br>9 w	trial 2<br>10 w	trial 3<br>11 w
Control	Mean	358.8	366.2	391.8
	±SD	57.2	66.1	73.0
	±SEM	20.2	23.4	25.8
	Median	353.3	364.3	392.5
	upper limit	463.0	480.0	480.0
	lower limit	287.0	284.3	285.0
	N (m/f)	8 (4/4)	8 (4/4)	8 (4/4)
TIF-IA^{D1Cre}	Mean	372.5	356.8	416.7
	±SD	90.1	119.4	58.8
	±SEM	36.8	48.8	24.0
	Median	380.2	347.0	424.0
	upper limit	470.0	513.3	480.0
	lower limit	242.0	167.3	313.0
	N (m/f)	6 (2/4)	6 (2/4)	6 (2/4)
R6/2	Mean	252.8	195.9	191.1
	±SD	87.0	69.0	70.8
	±SEM	25.1	19.9	20.4
	Median	242.7	202.2	188.3
	upper limit	413.7	296.0	283.3
	lower limit	140.0	72.0	52.0
	N (m/f)	12 (5/7)	12 (5/7)	12 (5/7)
dm	Mean	196.1	108.2	92.1
	±SD	61.5	33.6	33.4
	±SEM	17.8	9.7	9.6
	Median	200.7	115.5	96.0
	upper limit	293.0	162.5	141.0
	lower limit	92.7	46.3	36.5
	N (m/f)	12 (7/5)	12 (7/5)	12 (7/5)

*Two-way ANOVA	
Source of Variation	P value
Interaction	**0.0222**
Age	**0.0681**
Genotype	**<0.0001**

Results of Tukey's multiple comparison p value	trial 1	trial 2	trial 3
Control vs. TIF-IA^{D1Cre}	0.9824	0.9942	0.9069
Control vs. R6/2	**0.0053**	**<0.0001**	**<0.0001**
Control vs. dm	**<0.0001**	**<0.0001**	**<0.0001**
TIF-IA^{D1Cre} vs. R6/2	**0.0038**	**<0.0001**	**<0.0001**
TIF-IA^{D1Cre} vs. dm	**<0.0001**	**<0.0001**	**<0.0001**
R6/2 vs. dm	0.1836	**0.0117**	**0.0033**

E) Grip strength (mN)				
		trial 1<br>9 w	trial 2<br>10 w	trial 3<br>11 w
Control	Mean strength (mN)	616.7	641.4	652.7
	±SD	89.6	101.3	95.4
	±SEM	31.7	35.8	33.7
	Median	599.2	670.2	670.5
	upper limit	793.7	742.7	782.7
	lower limit	501.7	449.7	514.3
	N (m/f)	8 (4/4)	8 (4/4)	8 (4/4)
TIF-IA^{D1Cre}	Mean strength (mN)	693.5	627.7	660.3
	±SD	156.3	124.5	81.1
	±SEM	63.8	50.8	33.1
	Median	720.7	678.3	644.0
	upper limit	858.0	708.3	769.7
	lower limit	406.7	380.0	564.7
	N (m/f)	6 (2/4)	6 (2/4)	6 (2/4)
R6/2	Mean strength (mN)	700.6	640.2	580.6
	±SD	119.2	120.2	94.1
	±SEM	34.4	34.7	27.2
	Median	738.8	629.7	611.5
	upper limit	838.0	817.7	688.3
	lower limit	413.3	494.0	408.3
	N (m/f)	12 (5/7)	12 (5/7)	12 (5/7)
dm	Mean strength (mN)	616.4	415.2	333.3
	±SD	103.2	95.5	104.0
	±SEM	29.8	27.6	30.0
	Median	648.7	413.3	341.0
	upper limit	750.3	581.0	463.7
	lower limit	409.7	232.7	181.7
	N (m/f)	12 (7/5)	12 (7/5)	12 (7/5)

Two-way ANOVA	
Source of Variation	P value
Interaction	**0.0006**
Age	**0.0005**
Genotype	**<0.0001**

Results of Tukey's multiple comparison p values	trial 1	trial 2	trial 3
Control vs. TIF-IA^{D1Cre}	0.5484	0.9954	0.9992
Control vs. R6/2	0.3215	>0,9999	0.4581
Control vs. Dm	>0,9999	**<0,0001**	**<0,0001**
TIF-IA^{D1Cre} vs. R6/2	0.9992	0.9955	0.4497
TIF-IA^{D1Cre} vs. dm	0.4786	**0.0008**	**<0,0001**
R6/2 vs. dm	0.2245	**<0,0001**	**<0,0001**

Table 6

Data and statistics for graphs shown in **Fig. 3,** N: number of mice. * Statistical significance is shown according to the Mann-Whitney U test (MWU).

A) D2R qPCR						
		**3 mo**	**4 mo**	**5 mo**	**6 mo**	**10 mo**
**control**	**mean**	1	1	1	1	1
	**±SD**	0.34	0.34	0.23	0.38	0.29
	**±SEM**	0.14	0.11	0.09	0.12	0.13
	**median**	1.00	0.98	0.91	0.96	0.96
	**upper limit**	1.40	1.69	1.26	1.50	1.34
	**lower limit**	0.67	0.66	0.72	0.41	0.67
	**N**	6	9	7	10	5
**zQ175**	**mean**	1.25	0.72	0.63	0.51	0.58
	**±SD**	0.21	0.12	0.20	0.19	0.25
	**±SEM**	0.09	0.04	0.08	0.07	0.11
	**median**	1.28	0.77	0.59	0.48	0.61
	**upper limit**	1.45	0.81	0.99	0.84	0.91
	**lower limit**	0.93	0.46	0.43	0.22	0.26
	**N**	5	8	6	8	5
***MWU**		0.178	0.114	**0.014**	**0.012**	**0.032**
**t-test**		0.186	**0.037**	**0.010**	**0.005**	**0.036**

A) D1R qPCR						
		**3 mo**	**4 mo**	**5 mo**	**6 mo**	**10 mo**
**control**	**mean**	1	1	1	1	1
	**±SD**	0.19	0.39	0.17	0.47	0.19
	**±SEM**	0.08	0.13	0.07	0.15	0.09
	**median**	0.96	0.86	1.02	0.97	0.95
	**upper limit**	1.28	1.90	1.21	1.76	1.31
	**lower limit**	0.78	0.60	0.70	0.34	0.79
	**N**	6	9	7	10	5
**zQ175**	**mean**	0.96	0.97	0.89	0.66	0.75
	**±SD**	0.34	0.10	0.18	0.10	0.11
	**±SEM**	0.14	0.04	0.07	0.04	0.05
	**median**	0.88	0.96	0.90	0.66	0.68
	**upper limit**	1.55	1.10	1.18	0.85	0.89
	**lower limit**	0.56	0.83	0.66	0.52	0.64
	**N**	6	8	6	8	5
***MWU**		0.699	0.541	0.295	0.101	**0.032**
**t-test**		0.828	0.825	0.270	0.061	**0.038**

D) intranuclear mHTT inclusions (% nuclei)						
	**mean**	**±SD**	**±SEM**	**Median**	**95% CI**	**n/N**
**5 months**	33.29	17.36	7.765	36.11	11.74-54.85	409/5
**10 months**	76.89	6.242	2.791	78.61	69.14-84.64	416/5
**MWU**	**0.0079**					
**t-test**	**0.0007**					

D) mHTT inclusion area (µm²)						
	**mean**	**±SD**	**±SEM**	**Median**	**95% CI**	**n/N**
**5 months**	1.21	0.21	0.09	1.17	0.96-1.47	409/5
**10 months**	1.61	0.33	0.15	1.74	1.21-2.02	416/5
**MWU**		0.0952				
**t-test**		**0.0487**				

D) mHTT intensity nucleoplasm/ inclusion ratio						
	**mean**	**±SD**	**±SEM**	**Median**	**95% CI**	**n/N**
**5 months**	0.64	0.11	0.05	0.61	0.51-0.78	409/5
**10 months**	0.44	0.05	0.02	0.43	0.38-0.50	416/5
**MWU**		**0.0159**				
**t-test**		**0.0054**				

Table 7

Data and statistics for graphs shown in **Suppl. Figure 4**; N: number of mice. Statistical significance is shown according to the Mann-Whitney U test (MWU).

A) Body weight (% of control)							
	**Mean**	**±SD**	**±SEM**	**Median**	**95% CI**	**N**	**m/f**
**Control**	100	8.2	2.5	103.2	94.5-105.5	11	5/6
**zQ175**	96.9	7.6	2.1	96.9	92.3-101.5	13	6/7
**MWU**	0.3607						
**t-test**	0.3464						

B) Rotarod endurance (s)							
	**Mean**	**±SD**	**±SEM**	**Median**	**95% CI**	**N**	**m/f**
**Control**	381	83.97	26.55	406.3	320.9-441	10	4/6
**zQ175**	324.7	83.86	23.26	282	274-375.4	13	6/7
**MWU**	0.1346						
**t-test**	0.1259						

C) Grip strength (mN)							
	**mean**	**±SD**	**±SEM**	**Median**	**95% CI**	**N**	**m/f**
**control**	541.8	149.6	45.12	586.3	441.2-642.3	11	5/6
**zQ175**	525.1	104.2	28.9	524.7	462.1-588.1	13	6/7
**MWU**	0.4244						
**t-test**	0.7513						

Table 8

Data and statistics for graphs shown in **Fig. 4**, n: number of analysed striatal nuclei, N: number of mice. * Statistical significance is shown according to the Mann-Whitney U test (MWU).

5 months: % of nuclei with nucleolar NPM1								
	**mean**	**±SD**	**±SEM**	**median**	**95% CI**	**N/n**	***MWU**	**t-test**
**control**	66.3	10.3	3.9	67.0	56.7-75.8	7/724	**0.0205**	**0.0108**
**zQ175**	41.6	19.7	7.0	42.2	25.1-58.0	8/720		

5 months: % of nuclei with nucleolar NCL								
	**mean**	**±SD**	**±SEM**	**median**	**95% CI**	**N/n**	**MWU**	**t-test**
**control**	84.1	8.2	3.1	82.7	76.6-91.7	7/480	0.7551	0.7489
**zQ175**	85.5	4.6	2.1	85.5	79.7-91.3	5/409		

10 months: % of nuclei with nucleolar NPM1								
	**mean**	**±SD**	**±SEM**	**median**	**95% CI**	**N/n**	**MWU**	**t-test**
**control**	77.7	14.9	6.7	70.7	59.2-96.3	5/771	0.1508	0.0756
**zQ175**	60.6	11.4	5.1	61.6	46.4-74.8	5/749		

10 months: % of nuclei with nucleolar NCL								
	**mean**	**±SD**	**±SEM**	**median**	**95% CI**	**N/n**	**MWU**	**t-test**
**control**	80.5	11.0	6.3	76.7	53.2-107.8	3/416	>0.9999	0.9876
**zQ175**	80.4	8.0	3.6	80.7	70.5-90.2	5/387		

Table 9

Data and statistics for graphs shown in **Suppl. Figure 6**, n: number of analysed striatal cells, N: number of mice. * Statistical significance is shown according to the Mann-Whitney U test (MWU).

B) 47S1 pre-rRNA			
		**5 months**	**10 months**
**control**	**Mean (fold change relative to control)**	1	1
	**±SD**	0.19	0.22
	**±SEM**	0.07	0.09
	**Median**	0.96	0.95
	**upper limit**	1.28	1.41
	**lower limit**	0.77	0.82
	**N**	7	6
**zQ175**	**Mean (fold change relative to control)**	0.89	1.15
	**±SD**	0.25	0.11
	**±SEM**	0.10	0.06
	**Median**	0.84	1.14
	**Upper 95% CI**	1.23	1.29
	**Lower 95% CI**	0.51	1.03
	**N**	6	4
**MWU**		0.366	0.2571

t-test	0.3836	0.2476

C) 47S2 pre-rRNA			
		5 months	10 months
Control	Mean (fold change relative to control)	1	1
	±SD	0.39	0.42
	±SEM	0.15	0.17
	Median	1.11	0.84
	upper limit	1.37	1.77
	lower limit	0.29	0.65
	N	7	6
zQ175	Mean (fold change relative to control)	0.69	1.20
	±SD	0.28	0.56
	±SEM	0.13	0.25
	median	0.56	1.20
	Upper 95% CI	1.10	2.00
	Lower 95% CI	0.43	0.49
	N	5	5
MWU		0.8238	0.4286
t-test		0.8092	0.5228

D) 18S rRNA			
		5 months	10 months
Control	Mean (fold change relative to control)	1	1
	±SD	0.23	0.06
	±SEM	0.08	0.03
	Median	0.96	0.99
	upper limit	1.39	1.08
	lower limit	0.73	0.91
	N	8	6
zQ175	Mean (fold change relative to control)	1.00	1.07
	±SD	0.20	0.29
	±SEM	0.08	0.13
	median	0.98	0.95
	Upper 95% CI	1.29	1.39
	Lower 95% CI	0.74	0.81
	N	6	5
MWU		0.9497	0.7922
t-test		0.9748	0.558

F) pre-rRNA foci per cell			
Age		**5 months**	**10 months**
**Control**	**Mean (fold change relative to control)**	1	1
	**±SD**	0.12	0.21
	**±SEM**	0.04	0.08
	**median**	1.00	1.01
	**upper limit**	0.91	0.80
	**lower limit**	1.09	1.20
	**n**	7793	4841
	**N**	9	7
**zQ175**	**Mean (fold change relative to control)**	1.19	0.97
	**±SD**	0.23	0.15
	**±SEM**	0.09	0.06
	**median**	1.09	0.99
	**Upper 95% CI**	0.98	0.81
	**Lower 95% CI**	1.40	1.12
	**n**	6494	5001
	**N**	7	6
**MWU**		0.0907	0.6282
**t-test**		**0.0441**	0.771

G) 5.8S / 5S rRNA by Northern blot						
	**mean**	**±SD**	**±SEM**	**median**	**95% CI**	**N**
**Control**	1.119	0.3084	0.09751	1.036	0.898-1.34	10
**zQ175**	1.155	0.2881	0.1018	1.051	0.914-1.396	8
**MWU**	0.6965					
**t-test**	0.8057					

H) 47S1 pre-rRNA muscle			
		**5 months**	**10 months**
**control**	**Mean (fold change relative to control)**	1	1
	**±SD**	0.62	0.40
	**±SEM**	0.36	0.20
	**Median**	0.83	0.95
	**upper limit**	1.69	1.48
	**lower limit**	0.48	0.61
	**N**	3	4
**zQ175**	**Mean (fold change relative to control)**	0.83	0.79
	**±SD**	0.43	0.36
	**±SEM**	0.19	0.14
	**Median**	0.76	0.81
	**Upper 95% CI**	1.46	1.20
	**Lower 95% CI**	0.31	0.21
	**N**	5	7
**MWU**		0.786	0.649
**t-test**		0.667	0.401

I) 47S2 pre-rRNA muscle			
		**5 months**	**10 months**
**Control**	**Mean (fold change relative to control)**	1	1
	**±SD**	0.34	0.31
	**±SEM**	0.20	0.15
	**Median**	1.05	0.98
	**upper limit**	1.31	1.39
	**lower limit**	0.64	0.65
	**N**	3	4
**zQ175**	**Mean (fold change relative to control)**	0.96	0.54
	**±SD**	0.45	0.13
	**±SEM**	0.20	0.05
	**median**	0.85	0.53
	**Upper 95% CI**	1.58	0.70
	**Lower 95% CI**	0.38	0.32
	**N**	5	7
***MWU**		>0,9999	**0.012**
**t-test**		0.908	**0.007**

J) 18S rRNA muscle			
		**5 months**	**10 months**
**Control**	**Mean (fold change relative to control)**	1	1
	**±SD**	0.12	0.09
	**±SEM**	0.06	0.05
	**Median**	0.98	1.02
	**upper limit**	1.19	1.15
	**lower limit**	0.81	0.85
	**N**	4	4
**zQ175**	**Mean (fold change relative to control)**	1.16	1.03
	**±SD**	0.26	0.23
	**±SEM**	0.12	0.09
	**median**	1.25	1.08
	**Upper 95% CI**	1.48	1.24
	**Lower 95% CI**	0.84	0.82
	**N**	5	7
**MWU**		0.286	0.788
**t-test**		0.301	0.799

Table 10

Data and statistics for graphs shown in **Fig. 5B, C**, n: number of DAPI positive nuclei in mouse quadriceps, N: number of mice. * Statistical significance is shown according to the Mann-Whitney U test (MWU).

B) 5 months: % nuclei with nucleolar NPM1								
	**mean**	**±SD**	**±SEM**	**median**	**95% CI**	**N/n**	**MWU**	**t-test**
**Control**	33.91	4.90	2.45	35.47	26.13-41.70	4/269	0.4127	0.4862
**zQ175**	29.67	10.56	4.72	33.17	16.57-42.78	5/593		

B) 5 months: % nuclei with nucleolar NCL								
	**mean**	**±SD**	**±SEM**	**median**	**95% CI**	**N/n**	**MWU**	**t-test**
**Control**	55.90	15.74	9.09	53.29	16.79-95.00	3/234	>0.9999	0.7856
**zQ175**	59.33	13.04	7.53	63.04	26.94-91.72	3/250		

B) 10 months: % nuclei with nucleolar NPM1								
	**mean**	**±SD**	**±SEM**	**median**	**95% CI**	**N/n**	**MWU**	**t-test**
**Control**	44.57	12.58	6.291	48.74	24.55-64.59	4/516	0.2857	0.2698
**zQ175**	35.00	11.38	5.091	31.89	20.86-49.13	5/659		

B) 10 months % nuclei with nucleolar NCL								
	**mean**	**±SD**	**±SEM**	**median**	**95% CI**	**N/n**	**MWU**	**t-test**
**control**	53.54	7.16	3.58	54.38	42.15-64.94	4/750	0.7619	0.8672
**zQ175**	54.45	8.68	3.54	52.60	45.34-63.56	6/949		

C) 5 months: NPM1 signal area (µm²)								
	**mean**	**±SD**	**±SEM**	**Median**	**95% CI**	**N/n**	**MWU**	**t-test**
**control**	3.16	0.51	0.228	2.927	2.53-3.79	5/220	0.2857	0.3456
**zQ175**	3.52	0.56	0.280	3.417	2.63-4.41	4/218		

C) 5 months: NCL signal area (µm²)								
	**Mean**	**±SD**	**±SEM**	**median**	**95% CI**	**N/n**	**MWU**	**t-test**
**control**	6.76	1.18	0.68	6.43	3.83-9.70	3/234	0.7000	0.4397
**zQ175**	5.84	1.45	0.84	6.26	2.25-9.43	3/250		

C) 10 months NPM1 area (µm²)								
	**mean**	**±SD**	**±SEM**	**median**	**95% CI**	**N/n**	***MWU**	**t-test**
**Control**	4.01	0.89	0.45	3.91	2.59-5.43	4/269	**0.0381**	**0.0134**
**zQ175**	2.67	0.47	0.19	2.69	2.18-3.16	6/306		

C) 10 months NCL area (µm²)								
	**mean**	**±SD**	**±SEM**	**median**	**95% CI**	**N/n**	**MWU**	**t-test**
**Control**	5.66	1.17	0.59	5.24	3.80-7.52	4/269	0.4127	0.7006
**zQ175**	5.90	0.58	0.26	6.02	5.18-6.61	5/593		

Table 11

Data and statistics for graphs shown in **Fig. 6B, C**, n: number of DAPI positive muscle nuclei, N: number of control individuals and Huntington's disease (HD) patients. * Statistical significance is shown according to the Kruskal-Wallis test.

**B) % nuclei with nucleolar NPM1**						
	**Mean**	**±SD**	**±SEM**	**median**	**95% CI**	**N/n**
**control**	36.99	4.86	2.17	38.01	30.96-43.03	5/991
**pre-HD**	25.80	3.66	1.64	27.34	21.26-30.35	5/844
**early HD**	20.99	2.63	1.17	20.89	17.73-24.25	5/794
**Kruskal-Wallis test p=0.0001**						
**p values from Dunn's multiple comparison**<br>**control vs. pre-HD** 0.1431**, control vs. early HD 0.0027, pre-HD vs. early HD** 0.5373						

**B) % nuclei with nucleolar NCL**						
	**Mean**	**±SD**	**±SEM**	**Median**	**95% CI**	**N/n**
**control**	65.08	5.79	2.89	66.55	55.87-74.29	4/101
**pre-HD**	64.18	4.97	2.22	62.38	58.01-70.36	5/145
**early HD**	68.31	4.09	2.04	69.31	61.8-74.81	4/120
**Kruskal-Wallis test** p=0.4499						
**p values from Dunn's multiple comparison**<br>**control vs. pre-HD>**0.9999**, control vs. early HD>**0.9999**, pre-HD vs. early HD** 0.5793						

**C) NPM1 area (µm^2)**						
	**Mean**	**±SD**	**±SEM**	**median**	**95% CI**	**N/n**
**control**	3.54	0.70	0.31	3.47	2.66-4.41	5/991
**pre-HD**	2.77	0.38	0.17	2.86	2.31-3.24	5/844
**early HD**	2.32	0.34	0.15	2.45	1.90-2.74	5/794
**Kruskal-Wallis test p=0.0059**						
**p values from Dunn's multiple comparison**<br>**control vs. pre-HD** 0.4708**, control vs. early HD 0.0099, pre-HD vs. early HD** 0.3843						

**C) NCL area (µm^2)**						
	**Mean**	**±SD**	**±SEM**	**median**	**95% CI**	**N/n**
**control**	2.73	0.60	0.30	2.69	1.77-3.69	4/101
**pre-HD**	2.78	0.63	0.28	2.44	2.00-3.55	5/145
**early HD**	2.69	0.41	0.21	2.57	2.03-3.35	4/120
**Kruskal-Wallis test** p=0.8755						
**p values from Dunn's multiple comparison**<br>**control vs. pre-HD>**0.9999**, control vs. early HD>**0.9999**, pre-HD vs. early HD>**0.9999						

Table 12

Data and statistics for graphs shown in **Suppl. Figure 7**, n: number of DAPI positive nuclei in mouse quadriceps, N: number of mice. * Statistical significance is shown according to the Mann-Whitney U test (MWU) or Kruskal-Wallis test.

A) 5 months DAPI area (µm^2)								
	**mean**	**±SD**	**±SEM**	**Median**	**95% CI**	**N/n**	**MWU**	**t-test**
**control**	27.51	4.714	2.108	27.15	21.66-33.36	5/607	0.4127	0.4093
**zQ175**	25.03	3.428	1.714	24.49	19.58-30.49	4/545		

B) 10 months DAPI area (µm^2)								
	**mean**	**±SD**	**±SEM**	**Median**	**95% CI**	**N/n**	**MWU**	**t-test**
**control**	21.92	2.26	1.13	21.66	18.32-25.53	4/432	0.9143	0.7105
**zQ175**	22.65	3.28	1.34	22.63	19.21-26.09	6/681		

C) human muscle DAPI area (µm^2)						
	**mean**	**±SD**	**±SEM**	**median**	**95% CI**	**n/N**
**control**	21.25	2.85	1.27	21.2	17.72-24.79	328/5
**pre-HD**	23.89	7.38	3.3	21.4	14.72-33.05	270/5
**early HD**	25.77	3.74	1.67	27.42	21.12-30.42	249/5
***Kruskal-Wallis test** p=0.3304						
**p values from Dunn's multiple comparison** **control vs. pre-HD** >0,9999; **control vs. early HD** 0.4127; **pre-HD vs. early HD** 0.8665						

Table 13

Data and statistics for graphs shown in **Suppl. Figure 8B**, n: number of DAPI positive nuclei in mouse quadriceps, N: number of mice. * Statistical significance is shown according to the Mann-Whitney U test (MWU).

B) % nuclei with nucleolar NPM1								
	**mean**	**±SD**	**±SEM**	**Median**	**95% CI**	**N/n**	***MWU**	**t-test**
**control**	19.72	8.20	3.35	21.91	11.12-28.33	6/672	**0.0095**	0.0101
**TIF-IA^{D1Cre}**	35.87	6.05	3.02	36.11	26.25-45.49	4/449		

B) NPM1 area (µm^2)								
	**mean**	**±SD**	**±SEM**	**Median**	**95% CI**	**N/n**	***MWU**	**t-test**
**control**	2.50	0.53	0.24	2.50	1.84-3.17	5/131	**0.0317**	0.0171
**TIF-IA^{D1Cre}**	3.52	0.42	0.21	3.49	2.85-4.20	4/161		

Table 14

Data and statistics for graphs shown in **Suppl. Figure 9**, n: number of DAPI positive muscle nuclei, N: number of control individuals and Huntington's disease (HD) patients. * Statistical significance is shown according to the Kruskal-Wallis test.

**C) % nuclei with nucleolar NPM1**						
	**Mean**	**±SD**	**±SEM**	**median**	**95% CI**	**n/N**
**control**	30.98	4.45	1.99	30.03	25.46-36.5	991/5
**pre-HD**	22.77	3.61	1.61	23.02	18.29-27.25	844/5
**early HD**	17.55	1.35	0.60	17.02	15.88-19.23	794/5
**Kruskal-Wallis test p<0.0001**						
**p values from Dunn's multiple comparison:**<br>**control vs. pre-HD** 0.2691**, control vs. early HD 0.0021, pre-HD vs. early HD** 0.2691						

**E) NPM1 area (μm^2)**						
	**mean**	**±SD**	**±SEM**	**median**	**95% CI**	**n/N**
**control**	3.59	0.68	0.30	3.77	2.75-4.43	991/5
**pre-HD**	2.79	0.81	0.36	2.52	1.78-3.79	844/5
**early HD**	2.25	0.23	0.10	2.23	1.97-2.54	794/5
**Kruskal-Wallis test p=0.0236**						
**p values from Dunn's multiple comparison:**<br>**control vs. pre-HD** 0.4719; **control vs. early HD 0.0267**; **pre-HD vs. early HD** 0.6880						
